# Efficacy and safety of entecavir, peginterferon alfa-2b and GM-CSF combination therapy: the anchor randomized controlled trial

**DOI:** 10.1007/s12072-025-10977-2

**Published:** 2025-12-09

**Authors:** Di Wu, Da Huang, Shifang Peng, Yongping Chen, Fengchun Yang, Xiaoyun Zhang, Lei Fu, Lanman Xu, Jiaji Jiang, Qi Zheng, Xinyue Chen, Yali Liu, Xiaoguang Dou, Ke Ma, Dong Xi, Peng Wang, Li Sun, Ruoyi He, Yuchen Tian, Ping Yin, Weiming Yan, Meifang Han, Qin Ning

**Affiliations:** 1 Department of Infectious Diseases, Tongji Hospital, Tongji Medical College, State Key Laboratory for Diagnosis and Treatment of Severe Zoonotic Infectious Disease, Huazhong University of Science and Technology, Wuhan, China; 2 Department of Infectious Diseases, Xiangya Hospital, Central South University, Changsha, China; 3 Hepatology Diagnosis and Treatment Center, The First Affiliated Hospital of Wenzhou Medical University, Wenzhou, China; 4 Zhejiang Provincial Key Laboratory for Accurate Diagnosis and Treatment of Chronic Liver Diseases, Wenzhou, China; 5 Department of Infectious Diseases, The First Affiliated Hospital of Wenzhou Medical University, Wenzhou, China; 6 The Affiliated People’s Hospital of Ningbo University, Ningbo, Zhejiang China; 7 Liver Research Center, First Affiliated Hospital of Fujian Medical University, Fuzhou, China; 8 International Medical Department, Beijing Youan Hospital, Capital Medical University, Beijing, China; 9 Department of Infectious Diseases, Shengjing Hospital of China Medical University, Shenyang, China; 10 Xiamen Amoytop Biotech Co Ltd, Xiamen, China; 11 Department of Epidemiology and Biostatistics, School of Public Health, Tongji Medical College, Huazhong University of Science and Technology, Wuhan, China

**Keywords:** Chronic hepatitis B, Functional cure, Peg-IFN, HBsAg loss, Combination treatment

## Abstract

**Background and aim:**

Functional cure is considered the advanced treatment goal for patients with chronic hepatitis B (CHB). We aimed to evaluate hepatitis B surface antigen (HBsAg) loss rates after combination treatment of entecavir (ETV) and peginterferon alfa-2b (Peg-IFN) with or without granulocyte–macrophage colony-stimulating factor (GM-CSF).

**Methods:**

In this randomized controlled trial, virally suppressed patients undergoing nucleos(t)ide analogs with HBsAg < 3000 IU/mL were randomized 1:1:1 to receive either ETV for 96 weeks (E group), or 48 weeks of Peg-IFN + ETV, followed by 48-week Peg-IFN alone (EP group), or 48 weeks of Peg-IFN + ETV + GM-CSF, followed by 48-week Peg-IFN alone (EPG group). The primary outcome is HBsAg loss at week 96.

**Results:**

Among 249 patients (81 in E group, 83 in EP group and 85 in EPG group), EP group (30.12%, 25.30%) and EPG group (22.35%, 20.00%) achieved significantly higher HBsAg loss and HBsAg seroconversion rates than E group (0.00%, 0.00%; *p* < .0001, *p* < .0001) at week 96. At week 120, HBsAg loss was maintained in 22 of 25 patients (88.00%) in EP group and 15 of 19 (78.95%) in EPG group. During the 24-week off-treatment follow-up, late HBsAg loss occurred in one EP and three EPG patients. Multivariate analysis showed that age, baseline HBsAg level, and 24-week HBsAg decline correlated with HBsAg loss and HBsAg seroconversion at week 96. 24-week HBV RNA decline correlated with HBsAg loss. The HBsAg dynamics during therapy could predict HBsAg loss with an area under the curve of 0.944. More patients in EP group and EPG group experienced adverse events than E group.

**Conclusion:**

In virally suppressed patients with HBsAg level < 3000 IU/mL, ETV plus Peg-IFN significantly increased HBsAg loss and HBsAg seroconversion, whereas adding GM-CSF conferred no additional efficacy benefit. Age, early on-treatment HBsAg level and 24-week HBV RNA decline correlated with HBsAg loss.

This trial is registered at ClinicalTrials.gov, NCT02327416.

**Supplementary Information:**

The online version contains supplementary material available at 10.1007/s12072-025-10977-2.

## Introduction

Functional cure for chronic hepatitis B virus (HBV) infection defined as sustained off-treatment serum hepatitis B surface antigen (HBsAg) loss is associated with improved clinical outcomes and thereby represents the ideal treatment endpoint [1–3]. However, antiviral therapies including nucleos(t)ide analogs (NA) or peginterferon alfa (Peg-IFN) when used alone rarely achieve functional cure as both drugs do not directly target HBV covalently closed circular DNA (cccDNA). Combining these two agents may improve the chance of functional cure through integrating potent antiviral and immunomodulatory activities. Our series of randomized controlled trials have provided direct evidence that for virally suppressed patients, switching to Peg-IFN-based therapy led to significant improvement in HBsAg loss rates over continuous entecavir (ETV) monotherapy [4, 5]. Successful response to Peg-IFN was associated with early significant restoration of immune responses, and effective antiviral immunity is a crucial step toward achieving functional cure [6].

Current guidelines highlight that HBsAg loss, regardless of (HBsAg) seroconversion, is a key criterion for defining functional cure [2, 3, 7]. However, hepatitis B surface antibody (HBsAb) development represents clinical recovery and immunity against HBV. Most patients achieving sustained HBsAg loss after Peg-IFN and NA combination therapy had developed HBsAb [8]. HBsAb positivity might be a surrogate endpoint given its correlation with functional cure and reduced recurrence risk [9].

Granulocyte–macrophage colony-stimulating factor (GM-CSF) is a potent hematopoietic growth factor that can promote the generation and propagation of antigen presenting cells and prime cellular immune response [10]. It can be used as an immunostimulatory adjuvant to elicit immune response to vaccines [11, 12]. The potential of GM-CSF to treat chronic hepatitis B (CHB) has been investigated. The combination of GM-CSF and IFN-α was effective in non-responders to IFN-α monotherapy [13]. A large-scale prospective study is warranted to explore whether the addition of GM-CSF to Peg-IFN therapy could improve functional cure rates. Thus, we aimed to investigate rates of HBsAg loss and safety profiles of combination therapy with Peg-IFN and GM-CSF in NA experienced patients.

## Methods

### Study design and participants

The anchor study was an open-label, randomized controlled study conducted between Dec 2014 and Jan 2018, at 6 sites in China. Patients aged 18–65 years with CHB, who had been receiving NA treatment for at least 1 year prior to enrollment, with HBV DNA levels ≤ 1000 copies/mL and HBsAg levels < 3000 IU/mL were eligible. Patients receiving IFN within the previous 6 months prior to enrollment, or with evidence of cirrhosis, hepatocellular carcinoma, hepatic decompensation, or co-infection with other viruses (human immunodeficiency virus, hepatitis C virus, or hepatitis D virus) were excluded as were patients with abnormal laboratory parameters, including alanine aminotransferase (ALT) greater than 10 × the upper limit of normal (ULN), or total bilirubin > 2 × ULN, etc.

This study was approved by the Independent Ethics Committee and was conducted in accordance with Good Clinical Practice guidelines and the Declaration of Helsinki. Written informed consent was obtained from all patients before enrollment. This trial is registered at ClinicalTrials.gov, NCT02327416.

### Randomization

Participants were randomized 1:1:1 to three groups. We used interactive web response system for computer-generated randomization sequences with a predefined stratification scheme. Randomization was stratified by sex and HBeAg status prior to NA treatment.

### Procedures

**Fig. 1:**
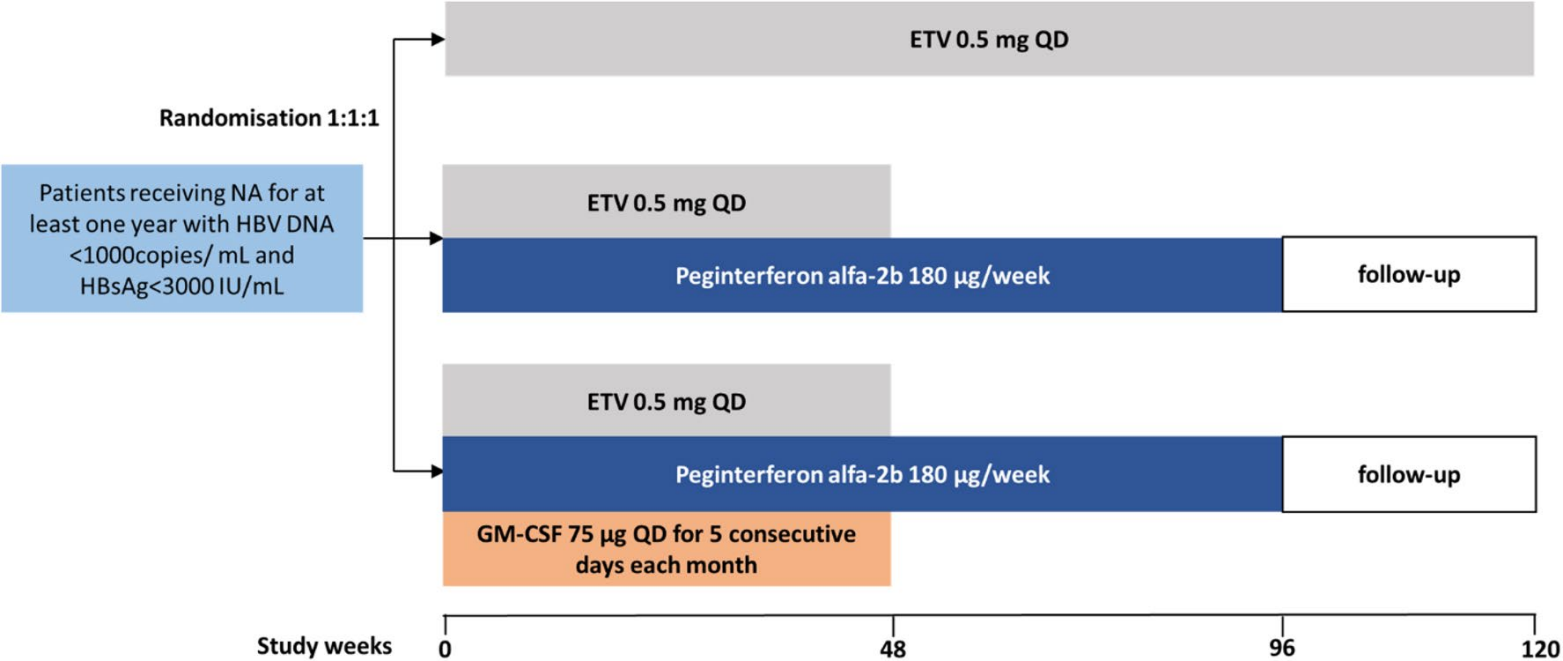
Trial design

As shown in Fig. 1, patients were randomly assigned to receive ETV monotherapy (Weiliqing, 0.5 mg once-daily; Jiangxi Qingfeng Pharmaceutical Co., Ltd, China) for 96 weeks (E group), or 48-week Peg-IFN alfa-2b (Paigebin, 180 mg once-weekly; Xiamen Amoytop Biotech Co., Ltd, China) and ETV combination therapy, followed by 48-week Peg-IFN alfa-2b monotherapy (EP group), or 48-week Peg-IFN alfa-2b, ETV, and GM-CSF (Teerli, 75 μg once-daily for 5 consecutive days each month; Xiamen Amoytop Biotech Co., Ltd, China) combination therapy, followed by 48-week Peg-IFN alfa-2b monotherapy (EPG group). All patients were followed up for 24 weeks. Patients in E group continued ETV during follow-up.

Routine biochemical and hematological tests were performed locally, virological assessments including HBV DNA, HBV RNA, HBsAg, HBsAb, and HBeAg quantifications were performed in centralized laboratory located at Tongji hospital, Wuhan, China. Serum HBV DNA (Roche COBAS AmpliPrep COBAS TaqMan HBVtest, v2.0 with an LLOD of 20 IU/mL), HBV RNA (HBV-SAT kit with LLOD of 50 copies/mL, the linear quantification range was 2–8 log_10_ copies/mL, Shanghai Rendu Biotechnology Co., Ltd. China), HBsAg (Roche Elecsys with LLOD of 0.05 IU/mL, Roche Diagnostics, Penzberg, Germany), and HBeAg (Architect HBeAg Reagent kit, HBeAg > 1.00 S/CO were defined as positive values, Abbott GmbH and Co. KG, Wiesbaden, Germany) and HBcrAg (LLOD of 2 log_10_U/mL, Fujirebio Inc, Tokyo, Japan) and HBsAb quantifications (Roche Elecsys Anti-HBs II assay with LLOD of 2 IU/L) were done during treatment and follow-up. Other laboratory analyses including urinalysis, thyroid function, coagulation test, antinuclear antibodies, alpha-fetoprotein and human chorionic gonadotropin, ultrasound, fibroscan, fundamental examination, etc. were assessed in accordance with the protocol.

Adverse events (AEs) and laboratory test results were recorded according to the International Conference on Harmonization Guideline Clinical Safety Data Management: Definitions and Standards for Expedited Reporting.

### Outcomes

The primary endpoint is HBsAg loss rates at week 96. Secondary efficacy endpoints included rates of HBsAg seroconversion (HBsAg loss and HBsAb level > 10 IU/L), and HBeAg loss, kinetics of HBsAg titers, rates of ALT normalization and HBV DNA < 20 IU/mL at week 96. Additionally, baseline and on-treatment factors associated with loss of 96-week HBsAg or HBsAg seroconversion were assessed. The safety endpoint was adverse events in three treatment groups. Adverse events were recorded at each visit. Safety assessments were done in the safety population.

### Statistical analysis

We used intention-to-treat (ITT) analysis as the primary analysis for all measures of efficacy or safety. As prespecified in the protocol, ITT analysis included all randomly allocated patients who received at least one dose of study drug except for those who withdrew consent or violated major eligibility criteria, with last observation carried forward for missing data.

Proportions were compared using a chi-squared test or Fisher’s exact test, and continuous variables were compared using two-way ANOVA or Kruskal–Wallis test as appropriate. All statistical tests were two-sided at 5% level of significance. We used exact logistic regression analysis to identify predictive factors of end-of-treatment HBsAg loss or HBsAb positivity. Univariates with a p value of < 0.05 were included in multivariable analysis. The longitudinal discriminant analysis algorithm based on the multiple linear mixed effects model was used to construct the prediction method for HBsAg loss as referred to a recent study [14]. The construction process of the model is depicted in Supplemental Material. The receiver operating characteristic (ROC) curve was used to evaluate the prediction accuracy of the model. The algorithm was implemented using the mixAK package (version 5.4). Statistical analysis was performed using SAS (version 9.4) and R (version 4.4.0).

## Results

### Baseline characteristics and patient disposition

**Fig. 2:**
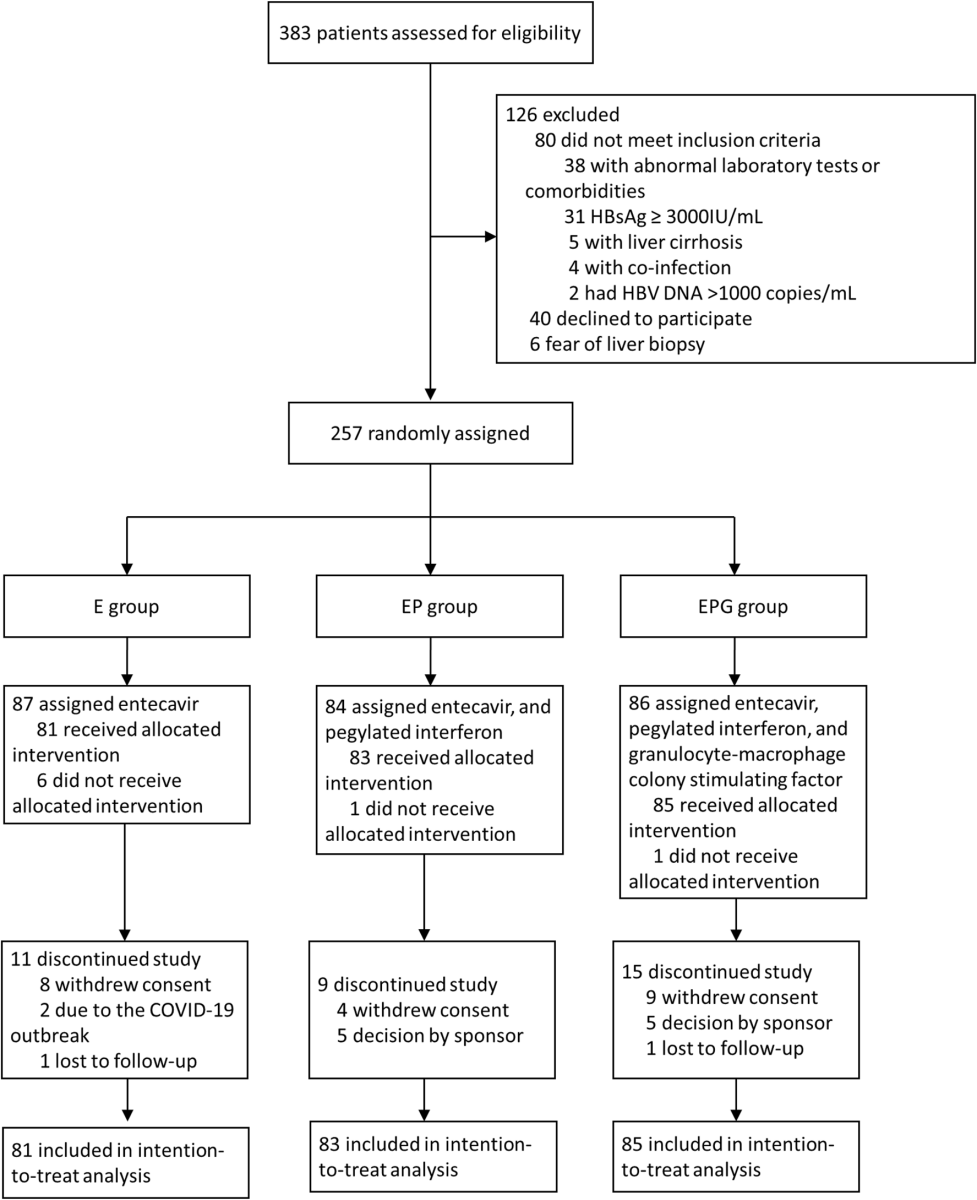
Trial flowchart

383 patients were screened, and 257 were randomized as depicted in Fig. 2. Of 257 patients randomized, 249 received at least one dose of the study drug (E group, n = 81; EP group, n = 83; EPG group, n = 85). 11 patients in E group, 9 in EP group and 15 in EPG group did not complete 96-week treatment. Baseline characteristics were comparable across three groups (Table 1).

**Table 1:** Baseline characteristics of participants among three groups

	E group (n = 81)	EP group (n = 83)	EPG group (n = 85)	p value
Age (years)	34.33 (20.07–59.65)	36.15 (18.34–52.94)	33.79 (20.83–51.81)	0.699
Sex				0.990
Male	68(83.95%)	69(83.13%)	71(83.53%)	
Female	13(16.05%)	14(16.87%)	14(16.47%)	
BMI (kg/m^2^)	22.76 (16.56–29.14)	22.68 (16.19–33.20)	22.56 (16.51–34.95)	0.927
HBeAg serology status prior to nucleos(t)ide analog				0.7856
Negative	22(27.16)	26(31.33)	23(27.06)	
Positive	59(72.84)	57(68.67)	62(72.94)	
HBeAg serology status at enrollment				0.582
Negative	43(53.09%)	51(61.45%)	46(54.12%)	
Positive	38(46.91%)	32(38.55%)	39(45.88%)	
HBsAg titers at enrollment (IU/mL)	975.65(859.20)	974.37(790.66)	897.81(741.79)	0.925
HBcrAg titers at enrollment (log_10_ U/mL)	4.91(1.25)	4.69(1.20)	4.79(1.16)	0.626
HBV RNA titers at enrollment (log_10_ copies/mL)	2.46(1.68)	2.37(1.88)	2.52(1.68)	0.882
Nucleos(t)ide analog treatment at entry				0.294
Entecavir	40(80.00%)	46(71.88%)	46(76.67%)	
Tenofovir	1(2.00%)	0(0.00%)	1(1.67%)	
Adefovir	7(14.00%)	8(12.50%)	6(10.00%)	
Lamivudine	1(2.00%)	4(6.25%)	0(0.00%)	
Telbivudine	1(2.00%)	6(9.38%)	7(11.67%)	
Two analogs	31(38.27%)	19(22.89%)	15(17.65%)	
Duration of nucleos(t)ide analog treatment (months)	64.27(14.00–175.00)	52.49(13.00–199.00)	57.78(13.00–169.00)	0.149
Previous interferon treatment*	12(14.81%)	15(18.07%)	11(12.94%)	0.646
ALT level (IU/L)	23.41(13.93)	24.66(13.54)	22.84(11.28)	0.647
AST level (IU/L)	21.65(7.25)	23.13(6.11)	21.59(5.41)	0.204
HBV genotype				0.279
B	2(2.47%)	23(27.7%)	26(30.59%)	
C	0(0.00%)	6(7.23%)	2(2.24%)	
Not available	79(97.53%)	54(65.06%)	57(67.06%)	

Data are median (IQR), n (%), or mean (SD)

BMI: body mass index; HBsAg: HBs antigen; HBV: hepatitis B virus; HBeAg: HBe antigen; ALT: alanine aminotransferase; AST: aspartate aminotransferase

^*^Any treatment with interferon in the past, with last intake less than 1 year before entry into the trial

### Treatment efficacy in the ITT population

**Fig. 3:**
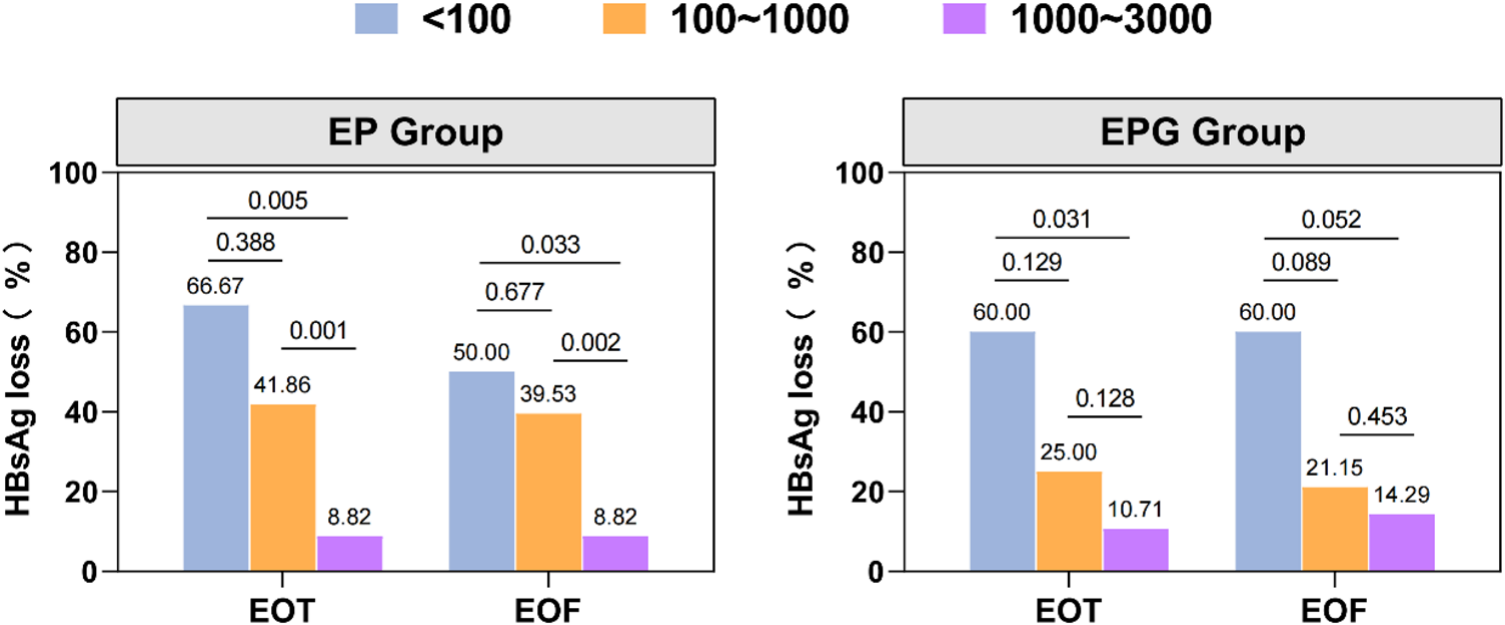
HBsAg loss rates at EOT and EOF stratified by baseline HBsAg levels in EP group and EPG group

EP group (30.12%, 27.71%) and EPG group (22.35%, 21.18%) achieved significantly higher rates of HBsAg loss than E group (0.00%, 0.00%) at week 96 (*p* < 0.0001, *p* < 0.0001, respectively, Table 2) and at week 120, while no significant difference was found between EP group and EPG group at week 96 or at week 120. We stratified patients by baseline HBsAg levels as follows: < 100 IU/mL, 100–1000 IU/mL, and 1000–3000 IU/mL. As illustrated in Fig. 3, patients with lower baseline HBsAg levels achieved higher HBsAg loss rates. 22 of 25 (88.00%) and 15 of 19 (78.95%) patients in EP group and EPG group, respectively, maintained HBsAg loss, while 1 of 58 (1.72%) and 3 of 66 (4.55%) patients who were HBsAg positive at week 96 in EP group and EPG group, respectively, achieved late HBsAg loss during a 24-week follow-up.

**Table 2:** Treatment responses among different groups

	E group	EP group	EPG group	p value		
				E group vs EP group	E group vs EPG group	EP group vs EPG group
HBsAg loss						
week 48	0/81 (0.00%)	17/83 (20.48%)	9/85 (10.59%)	< .0001	0.0026	0.0763
Difference in proportions (95% CI)				−20.48% (−29.16, −11.80)	−10.59% (−17.13, −4.05)	9.89% (−0.98, 20.76)
week 96	0/81 (0.00%)	25/83 (30.12%)	19/85 (22.35%)	< .0001	< .0001	0.2523
Difference in proportions (95% CI)				−30.12(−39.99, −20.25)	−22.35(−31.21,−13.50)	7.77(−5.49,21.03)
week 120	0/81 (0.00%)	23/83 (27.71%)	18/85 (21.18%)	< .0001	< .0001	0.3242
Difference in proportions (95% CI)				−27.71% (−37.34, −18.08)	−21.18% (−29.86, −12.49)	6.53% (−6.43, 19.50)
HBsAg seroconversion						
week 48	0/81(0.00%)	13/83(15.66%)	6/85 (7.06%)	0.0002	0.0149	0.0783
Difference in proportions (95% CI)				−15.66(−23.48,−7.84)	−7.06(−12.50,−1.61)	8.60(−0.92,18.13)
week 96	0/81(0.00%)	21/83(25.30%)	17/85(20.00%)	< .0001	< .0001	0.4116
Difference in proportions (95% CI)				−25.30(−34.65,−15.95)	−20.00(−28.50,−11.50)	5.30(−7.34,17.94)
week 120	0/81(0.00%)	17/83(20.48%)	16/85(18.82%)	< .0001	< .0001	0.7868
Difference in proportions (95% CI)				−20.48(−29.16,−11.80)	−18.82(−27.13,−10.51)	1.66(−10.36,13.68)
HBeAg loss*						
week 48	4/38(10.53%)	5/32(15.63%)	8/39(20.51%)	0.5255	0.2271	0.5962
Difference in proportions (95% CI)				−5.10(−21.02,10.82)	−9.99(−25.98,6.01)	−4.89(−22.74,12.97)
week 96	8/38(21.05%)	11/32(34.38%)	12/39(30.77%)	0.2118	0.3310	0.7467
Difference in proportions (95% CI)				−13.32(−34.27,7.63)	−9.72(−29.15,9.72)	3.61(−18.32,25.53)
week 120	6/38(15.79%)	13/32(40.63%)	16/39(41.03%)	0.0199	0.0143	0.9727
Difference in proportions (95% CI)				−24.84(−45.43,−4.24)	−25.24(−44.54,−5.93)	−0.40(−23.38,22.57)
HBV DNA < 20 IU/mL						
week 48	76/81(93.83%)	74/83(89.16%)	77/85(90.59%)	0.2845	0.4375	0.7584
Difference in proportions (95% CI)				4.67(−3.83,13.17)	3.24(−4.89,11.36)	−1.43(−10.56,7.69)
week 96	77/81(95.06%)	69/83(83.13%)	67/85(78.82%)	0.0145	0.0020	0.4770
Difference in proportions (95% CI)				11.93(2.59,21.27)	16.24(6.35,26.12)	4.31(−7.54,16.16)
week 120	76/81(93.83%)	61/83(73.49%)	48/85(56.47%)	0.0004	< .0001	0.0208
Difference in proportions (95% CI)				20.33(9.49,31.18)	37.36(25.59,49.13)	17.02(2.84,31.21)
HBV DNA > 200 IU/mL during follow−up						
week 96	2/81(2.47%)	6/83(7.23%)	10/85(11.76%)	0.1571	0.0208	0.3167
Difference in proportions (95% CI)				−4.76(−11.28,1.76)	−9.30(−16.93,−1.66)	−4.54(−13.36,4.29)
week 120	3/81(3.70%)	10/83(12.05%)	23/85(27.06%)	0.0480	< .0001	0.0144
Difference in proportions (95% CI)				−8.34(−16.47,−0.22)	−23.36(−33.66,−13.05)	−15.01(−26.77,−3.25)
ALT normalization						
week 48	78/81(96.30%)	52/83(62.65%)	53/85(62.35%)	< .0001	< .0001	0.9682
Difference in proportions (95% CI)				33.65(22.46,44.84)	33.94(22.85,45.03)	0.30(−14.34,14.94)
week 96	76/81(93.83%)	59/83(71.08%)	59/85(69.41%)	0.0001	< .0001	0.8126
Difference in proportions (95% CI)				22.74(11.67,33.82)	24.42(13.31,35.52)	1.67(−12.15,15.50)
week 120	74/81(91.36%)	69/83(83.13%)	77/85(90.59%)	0.1150	0.8627	0.1521
Difference in proportions (95% CI)				8.23(−1.89,18.34)	0.77(−7.95,9.49)	−7.46(−17.63,2.71)

Data are n/N (%) unless otherwise stated

E: entecavir; EP: entecavir, and pegylated interferon; EPG: entecavir, pegylated interferon, and granulocyte–macrophage colony-stimulating factor; HBsAg: HBs antigen; HBsAb: anti-HBs antibody; HBV: hepatitis B virus; HBeAg: HBe antigen; ALT: alanine aminotransferase

^*^Among patients who were HBeAg-positive at enrollment

Likewise, the rates of HBsAg seroconversion were significantly higher in EP group (25.30%, 20.48%) and EPG group (20.00%, 18.82%) than in E group (0.00%, 0.00%) at week 96 (*p* < 0.0001, *p* < 0.0001, respectively) and week 120, while no significant difference was found between EP group and EPG group at week 96 or 120.

Among the patients who were HBeAg-positive at baseline, 21.05%, 34.38%, and 30.77% in E group, EP group and EPG group, respectively, achieved HBeAg loss at week 96, and no significant difference was observed among the three groups. HBeAg loss rates in both EP and EPG groups continued to increase through week 120. Significantly more patients in EP group (40.63%) and EPG group (41.03%) achieved HBeAg loss than in E group (15.79%) at week 120 (p = 0.0199, p = 0.0143, respectively), while no significant difference was found between EP group and EPG group at week 120.

The HBV DNA undetectable rates were significantly higher in E group (95.06%) than that in EP group (83.13%) and EPG group (78.82%) at week 96 (p = 0.0145, p = 0.0020, respectively), while no significant difference was found between the latter two groups (p = 0.4770). At week 120, 93.83% of patients in E group had undetectable HBV DNA, which was higher than EP group (73.49%; *p* = 0.0004) and EPG group (56.47%; *p* < 0.0001). EPG group had significantly lower rate of HBV DNA suppression than EP group (*p* = 0.0208).

### Longitudinal analysis of virological and biochemical markers across three groups

**Fig. 4:**
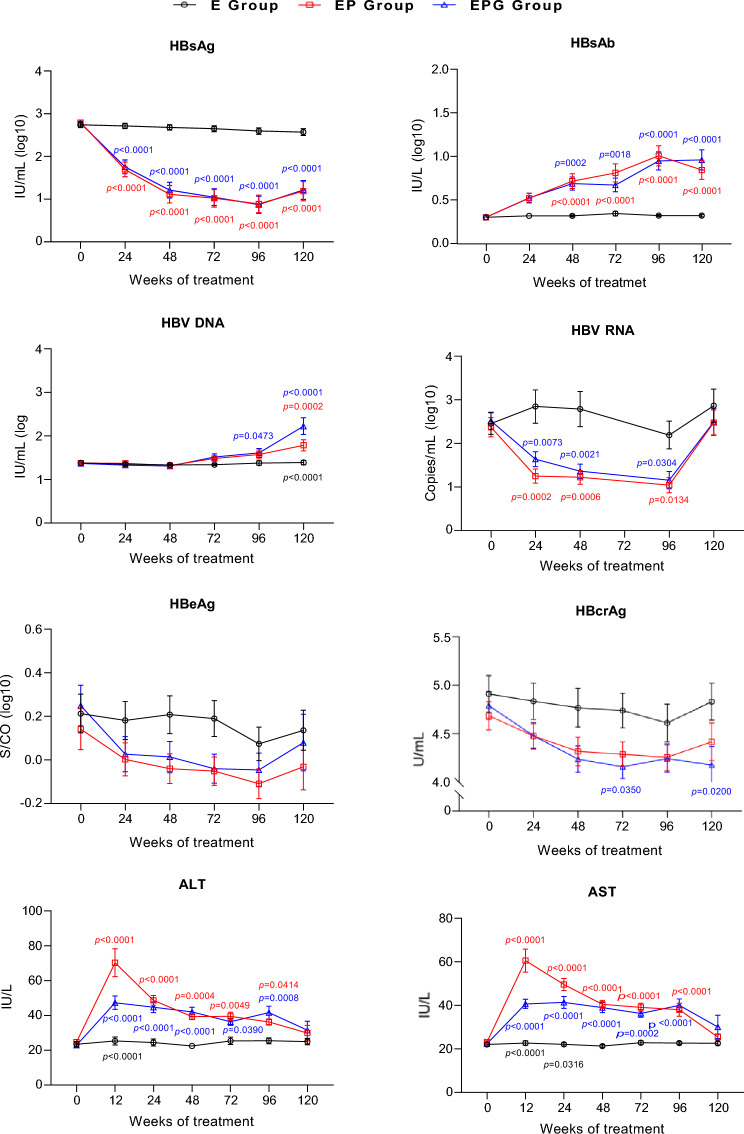
Longitudinal changes in virological and biochemical markers among groups. Mean levels of HBsAg, HBsAb, HBV DNA, HBV RNA, HBeAb, HBcrAg, ALT and AST. Blue: EPG group vs E group; Red: EP group vs E group; Black: EP group vs EPG group

As shown in Fig. 4, EP and EPG group exhibited significantly lower HBsAg levels but higher HBsAb levels than E group after week 24. Similarly, profoundly lower HBeAg and HBcrAg levels at week 48 and 72, HBV RNA at week 24, 48, and 96 were observed in EP and EPG group. After NA cessation at week 48, HBV DNA levels gradually increased in EP and EPG group, which were significantly higher than that in E group at week 72, 96, and 120. After initiating Peg-IFN, some patients particularly those in EP group experienced ALT elevation, and mean ALT levels significantly increased during Peg-IFN-based therapy at week 12, then declined gradually. Longitudinal FIB-4 indices and fibroscan measurements are shown in Supplementary Fig. 1.

### Baseline and on-treatment predictors of HBsAg loss or HBsAg seroconversion at week 96 in patients receiving Peg-IFN-based therapy

As shown in Table 3, univariate analysis revealed that age and early on-treatment levels of HBsAg, HBcrAg, HBV RNA, and HBsAb were associated with HBsAg loss or HBsAg seroconversion at week 96 in patients receiving Peg-IFN-based therapy (both EP group and EPG group). In multivariate analysis, age (p = 0.027, p = 0.016, respectively), baseline HBsAg level (p = 0.001, p = 0.001, respectively), and HBsAg decline at week 24 (p = 0.004, p = 0.002, respectively) were independently associated with HBsAg loss or HBsAg seroconversion at week 96. HBV RNA decline at week 24 (p = 0.010) correlated with HBsAg loss at week 96, while HBsAb level at week 24 (p = 0.031) correlated with HBsAg seroconversion at week 96.

**Table 3:** Baseline and on-treatment predictors of HBsAg loss and HBsAb seroconversion in patients treated with Peg-IFN-based therapy*

	HBsAg loss				HBsAb seroconversion			
	Univariate Analysis OR (95%CI)	*p*	Multivariate Analysis OR (95%CI)	*p*	Univariate Analysis OR (95%CI)	*p*	Multivariate Analysis OR (95%CI)	*p*
Group								
EP group	1.000 (Reference)				1.000 (Reference)			
EPG group	0.668 (0.334 ~ 1.336)	0.254			0.738 (0.357 ~ 1.526)	0.412		
Sex								
Male	1.000 (Reference)				1.000 (Reference)			
Female	1.156 (0.468 ~ 2.852)	0.754			1.467 (0.588 ~ 3.658)	0.411		
Age, years	0.949 (0.910 ~ 0.989)	**0.013**	0.898 (0.817 ~ 0.988)	**0.027**	0.934 (0.893 ~ 0.978)	**0.003**	0.897 (0.822 ~ 0.980)	**0.016**
HBeAg status								
Positive	1.000 (Reference)				1.000 (Reference)			
Negative	1.078 (0.536 ~ 2.168)	0.833			0.878 (0.424 ~ 1.818)	0.726		
HBV DNA status								
Positive	1.000 (Reference)				1.000 (Reference)			
Negative	0.985 (0.333 ~ 2.918)	0.979			1.095 (0.340 ~ 3.525)	0.879		
HBV RNA status								
Positive	1.000 (Reference)				1.000 (Reference)			
Negative	1.180 (0.563 ~ 2.471)	0.661			1.267 (0.583 ~ 2.752)	0.550		
qHBsAg, log10 IU/mL								
Baseline	0.177 (0.079 ~ 0.400)	** < .001**	0.037 (0.005 ~ 0.281)	**0.001**	0.238 (0.107 ~ 0.531)	** < .001**	0.048 (0.007 ~ 0.312)	**0.001**
Week 12	0.175 (0.096 ~ 0.319)	** < .001**			0.244 (0.144 ~ 0.411)	** < .001**		
Week 24	0.295 (0.204 ~ 0.426)	** < .001**			0.353 (0.254 ~ 0.489)	** < .001**		
qHBsAg decline from baseline, log_10_ IU/mL								
Week 12	6.255 (3.146 ~ 12.439)	** < .001**			4.889 (2.663 ~ 8.974)	** < .001**		
Week 24	3.589 (2.430 ~ 5.300)	** < .001**	2.433 (1.327 ~ 4.459)	**0.004**	3.127 (2.170 ~ 4.506)	** < .001**	2.353 (1.361 ~ 4.069)	**0.002**
qHBeAg, log10 S/CO								
Baseline	0.928 (0.617 ~ 1.398)	0.722			0.948 (0.617 ~ 1.455)	0.806		
Week 24	0.698 (0.399 ~ 1.220)	0.207			0.658 (0.361 ~ 1.196)	0.170		
qHBeAg decline from baseline, log_10_ S/CO								
Week 24	1.939 (0.824 ~ 4.560)	0.129			2.077 (0.871 ~ 4.950)	0.099		0.441
qHBcrAg, log10 U/mL								
Baseline	0.712 (0.508 ~ 0.998)	**0.049**	0.739 (0.325 ~ 1.683)	0.472	0.780 (0.551 ~ 1.105)	0.162		
Week 12	0.767 (0.534 ~ 1.100)	0.149			0.846 (0.582 ~ 1.229)	0.380		
Week 24	0.608 (0.412 ~ 0.896)	**0.012**			0.652 (0.438 ~ 0.970)	**0.035**		
qHBcrAg decline from baseline, log_10_ U/mL								
Week 12	0.530 (0.180 ~ 1.559)	0.249			0.594 (0.207 ~ 1.710)	0.334		
Week 24	1.798 (0.720 ~ 4.491)	0.209	0.242 (0.039 ~ 1.484)	0.125	2.418 (0.940 ~ 6.218)	0.067	0.352 (0.070 ~ 1.767)	0.929
qHBV DNA, log_10_ IU/mL								
Baseline	0.857 (0.225 ~ 3.268)	0.821			0.859 (0.208 ~ 3.545)	0.833		
Week 12	1.257 (0.416 ~ 3.797)	0.685			1.006 (0.293 ~ 3.456)	0.992		
Week 24	2.488 (0.181 ~ 34.207)	0.496			1.328 (0.078 ~ 22.658)	0.845		
qHBV RNA, log_10_ copies/mL								
Baseline	1.002 (0.814 ~ 1.235)	0.982			1.006 (0.808 ~ 1.252)	0.959		
Week 12	0.560 (0.392 ~ 0.802)	**0.002**			0.585 (0.403 ~ 0.849)	**0.005**		
Week 24	0.336 (0.216 ~ 0.524)	** < .001**			0.329 (0.204 ~ 0.532)	** < .001**		
qHBV RNA decline from baseline, log_10_ copies/mL								
Week 12	1.657 (1.198 ~ 2.292)	**0.002**			1.505 (1.093 ~ 2.073)	**0.012**		
Week 24	1.771 (1.344 ~ 2.334)	** < .001**	2.066 (1.192 ~ 3.583)	**0.010**	1.674 (1.276 ~ 2.196)	** < .001**	1.510 (0.933 ~ 2.443)	0.191
qHBsAb, log_10_ IU/L								
Week 24	5.991 (2.581 ~ 13.903)	** < .001**	2.239 (0.583 ~ 8.597)	0.240	5.957 (2.621 ~ 13.538)	** < .001**	2.335 (0.715 ~ 7.629)	**0.031**
ALT, IU/L								
Baseline	1.003 (0.976 ~ 1.031)	0.826			0.995 (0.966 ~ 1.026)	0.766		
Week 12	1.008 (0.999 ~ 1.016)	0.067			1.006 (0.999 ~ 1.014)	0.095		
Week 24	1.001 (0.987 ~ 1.014)	0.936			1.000 (0.986 ~ 1.015)	0.946		

OR: Odds Ratio; CI: Confidence Interval; HBsAg: HBs antigen; HBsAb: anti-HBs antibody; HBV: hepatitis B virus; HBeAg: HBe antigen; NA: nucleos(t)ide analogs; ALT: alanine aminotransferase

^*^Included the patients in EP group and EPG group

### HBsAg dynamics may predict HBsAg loss

**Fig. 5:**
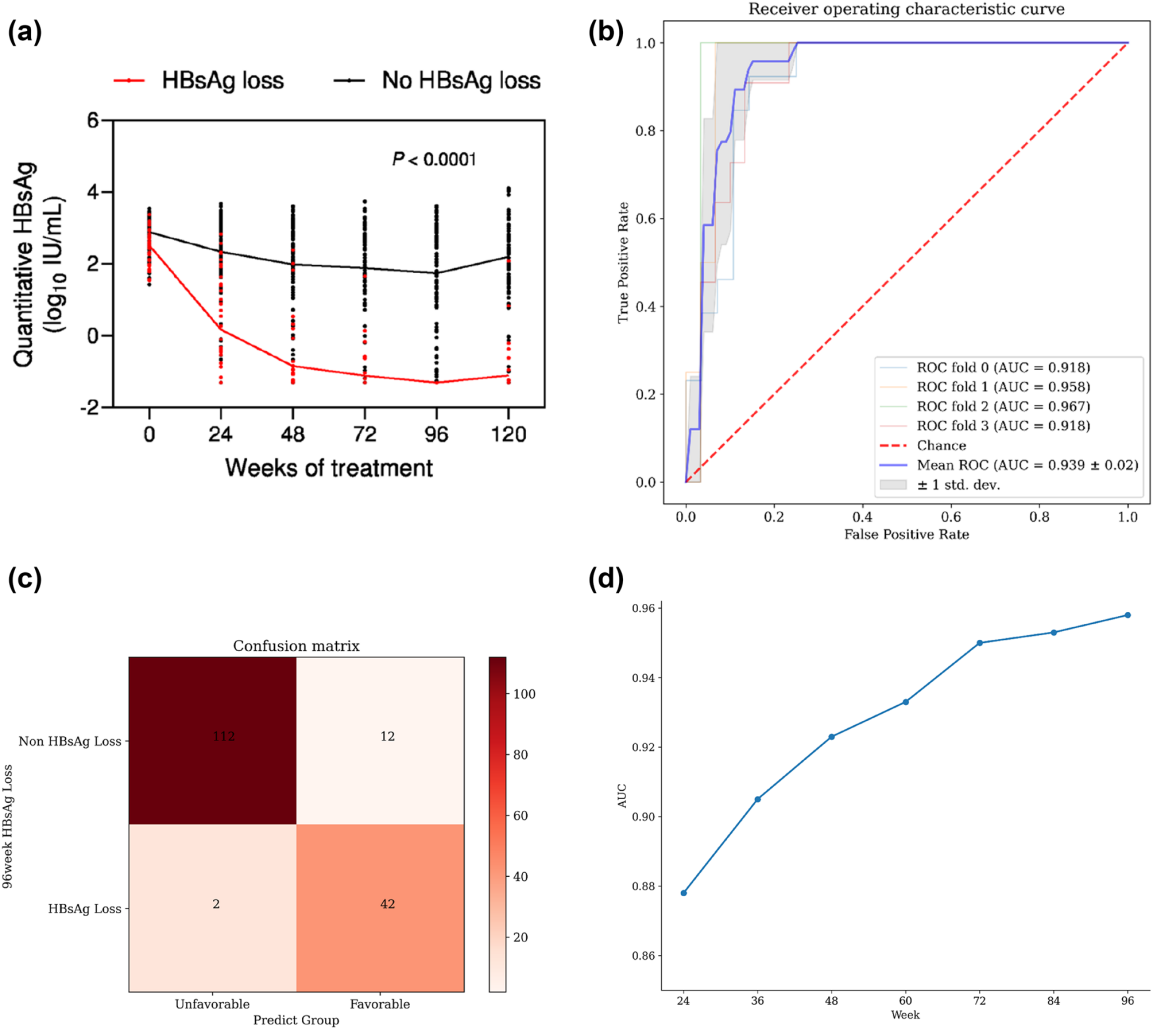
The HBsAg dynamics and the predictive performance of the LoAD model to predict HBsAg loss. **A** HBsAg levels in patients with and without HBsAg loss. Levels of significance: *p* < 0.0001. **B** The AUC of HBsAg longitudinal model under four-fold cross-validation. **C** The confusion matrix evaluating the performance of the model. **D** The performance of model involving varying numbers of timepoints of HBsAg values

The dynamics of HBsAg levels exhibited distinct patterns between patients with and without HBsAg loss (Fig. 5A). During treatment, HBsAg levels declined rapidly in patients with HBsAg loss, but declined slightly in those without HBsAg loss. A model using serial HBsAg measurements categorizes patients into the unfavorable group and the favorable group. The mean area under the receiver operating characteristic curve (AUROC) was 0.939 (95%CI 0.899–0.982) (Fig. 5B). The confusion matrix was employed to assess the model’s performance. Across the entire population, the model demonstrated an accuracy of 91.7%. Within the favorable group, 77.8% of patients achieved HBsAg loss. In the unfavorable group, 98.2% of patients did not achieve HBsAg loss. The model performed well in both groups separately (Supplementary Fig. 2A and 2B). Additionally, the AUC values increased gradually as more time-points were incorporated for prediction (Fig. 4D).

### Safety

The safety profiles of the three treatment arms are summarized in Table 4. More patients in EP group (95.18%, 95.18%) and EPG group (95.29%, 94.12%) than E group (70.37%, 17.28%) experienced AEs and drug-related AEs respectively. Five patients in EP group and 5 in EPG group discontinued treatment for safety reasons. 4 patients in EP group and 1 patient in EPG group reported serious AEs related to therapy. In EP group, one patient experienced ALT level > 10 × ULN, and another patient had perianal abscess. Both conditions resolved after discontinuation of Peg-IFN therapy. One patient in EPG group had diabetes. The common side events in EP group and EPG group were those known to occur with Peg-IFN therapy, including pyrexia, fatigue, dizziness, myalgia, headache, injection site reaction, appetite loss, alopecia, etc. 6 patients in EP group and 12 patients EPG group had retinal hemorrhage. The most frequent laboratory abnormalities during Peg-IFN-based therapy included neutropenia, leukopenia, thrombocytopenia, ALT and AST elevation. The incidence of neutropenia in EP group (51.81%) was higher than EPG group (36.47%), whereas the incidence of pyrexia and headache in EP group (78.31%, 36.14%) was lower than EPG group (90.59%, 54.12%). In EP group, 36 patients reduced their doses of Peg-IFN during the treatment period, with 13 reductions due to neutropenia. In EPG group, 29 patients reduced their doses, with 7 reductions due to neutropenia. Additionally, 14 patients in EP group and 16 in EPG group held Peg-IFN therapy, with 1 patient in each group discontinuing treatment due to neutropenia.

**Table 4:** Adverse events and laboratory abnormalities from week 0 to week 120

	E group (n = 81)		EP group(n = 83)		EPG group (n = 85)	
	AEs	Patients	AEs	Patients	AEs	Patients
All AEs						
Total AEs	304	57 (70.37%)	1311	79 (95.18%)	1664	81(95.29%)
AEs related to therapy	27	14 (17.28%)	990	79(95.18%)	1406	80(94.12%)
Serious AEs	11	3(3.70%)	16	7(8.43%)	4	4(4.71%)
Serious AEs related to therapy	0	0(0.00%)	4	3(3.61%)	1	1(1.18%)
Most frequent events *						
Pyrexia	14	12(14.81%)	148	65(78.31%)	251	77(90.59%)
Fatigue	9	8(9.88%)	89	51(61.45%)	143	48(56.47%)
Dizziness	7	5(6.17%)	57	33(39.76%)	80	30(35.29%)
Myalgia	2	2(2.47%)	63	30(36.14%)	82	40(47.06%)
Headache	11	10(12.35)	76	30(36.14%)	138	46(54.12%)
Injection site reaction	1	1(1.23%)	31	20(24.10%)	60	24(28.24%)
Loss of appetite	0	0(0.00%)	28	20(24.10%)	38	20(23.53%)
Alopecia	0	0(0.00%)	23	20(24.10%)	18	13(15.29%)
Pruritus	1	1(1.23)	19	15(18.07%)	15	11(12.94%)
Nausea	3	2(2.47%)	10	9(10.84%)	19	10(11.76%)
Cough	16	11(13.58%)	11	8(9.64%)	23	21(24.71%)
Rash	0	0(0.00%)	18	9(10.84%)	28	17(20.00%)
Arthralgia	1	1(1.23%)	27	9(10.84%)	25	15(17.65%)
Diarrhea	5	4(4.94%)	9	8(9.64%)	24	16(18.82%)
Vomiting	0	0(0.00%)	13	9(10.84%)	8	6(7.06%)
Retinal hemorrhage	0	0(0.00%)	7	6(7.23%)	13	12(14.12%)
Most frequent laboratory abnormalities*						
Neutropenia	0	0(0.00%)	60	43(51.81%)	45	31(36.47%)
Leukopenia	1	1(1.23%)	42	25(30.12%)	29	19(22.35%)
Thrombocytopenia	2	2(2.47%)	27	22(26.51%)	18	16(18.82%)
ALT elevation	7	4(4.94%)	44	32(38.55%)	51	31(36.47%)
AST elevation	2	2(2.47%)	36	29(34.94%)	40	30(35.29%)

Data are n or n (%)

AE: adverse event; ALT: alanine aminotransferase; AST: aspartate aminotransferase

^*^Events occurring in more than 10% of patients

## Discussion

To our knowledge, this is the first randomized controlled trial evaluating HBsAg loss and HBsAb response of adding Peg-IFN and GM-CSF to a stable NA regimen. Add-on Peg-IFN therapy demonstrated relatively high rates of HBsAg loss (30.12%), which were higher than that reported in previous trials [4, 15]. This may be partly attributed to low baseline HBsAg levels and extended duration of Peg-IFN treatment. After a 24-week follow-up, HBsAg loss was sustained in the majority of responders. Moreover, HBsAb positivity was observed in roughly one-third of patients. GM-CSF, when added to Peg-IFN-based therapy, failed to improve HBsAg loss rates or any secondary efficacy endpoints, and although it offered protection against neutropenia, routine use of GM-CSF with Peg-IFN is not recommended. The role of HBsAg levels in predicting HBsAg loss has been widely recognized. Here we reported that in addition to age, early on-treatment HBsAg level or decline, 24-week HBV RNA decline may predict end-of-treatment HBsAg loss, while early HBsAb appearance was associated with end-of-treatment HBsAg seroconversion. Notably, HBsAg dynamics during therapy was identified as a strong predictor of HBsAg loss. These findings may help optimize therapeutic strategies toward functional cure of CHB and early identify candidates suitable for Peg-IFN-based treatment.

Add-on of Peg-IFN to a stable NA regimen has proven effective in improving functional cure rates than continuous NA therapy [4, 5]. NA-treated patients with maintained viral suppression and low HBsAg levels are most likely to benefit from Peg-IFN treatment, either in a “switch-to” or “add-on” manner [16]. However, the “switch-to” strategy may carry high risk of relapse after NA cessation [17], we therefore adopted a Peg-IFN “add-on” strategy, using ETV and Peg-IFN concomitantly for 48 weeks, followed by Peg-IFN alone for additional 48 weeks to consolidate the response. Adding extended duration of Peg-IFN therapy significantly improved the rates of HBsAg loss and HBsAg seroconversion than ETV monotherapy in patients with baseline HBsAg < 3000 IU/mL. The responses continued to improve from week 48 to week 96; thus, the prolonged immunomodulator may enable more appropriate patients to attain functional cure. Intriguingly, the simultaneous presence of HBsAg and HBsAb was observed during Peg-IFN-based therapy. At the end of treatment (EOT), 16 patients were HBsAb-positive without HBsAg loss. Among them, only one patient whose HBsAg level was 0.114 IU/mL at EOT cleared HBsAg during the 24-week follow-up. In the remaining 15 patients, HBsAg levels generally rebounded after Peg-IFN was discontinued. These findings suggest that durable immune control of HBV is indicated not merely by detectable HBsAb, but specifically by HBsAg seroconversion.

Immune restoration is the crucial path to achieving functional cure. Long-term NA therapy can partially recover HBV-specific T cells function [18]. Under this scenario, additional Peg-IFN will activate the immune response [19], subsequently leading to HBsAg clearance. We speculate that adding immunomodulators may improve the chance of functional cure for CHB. GM-CSF is a potent cytokine that can promote both humoral and cellular immunity [10]. Animal studies suggested that combining GM-CSF and a recombinant HBV vaccine with or without IFN-α led to HBeAg and HBsAg clearance [20, 21]. In the present study, however, the addition of GM-CSF to Peg-IFN demonstrated no benefit for HBsAg loss, HBeAg loss or HBV DNA suppression. Given that GM-CSF promotes the function of antigen presenting cells, it has been used as an immunostimulatory adjuvant in vaccines to elicit immune response [11, 12]. GM-CSF as adjuvant to hepatitis B vaccine can improve HBsAb response in healthy subjects [22]. Consistently, a recent study demonstrated that in patients receiving NA treatment, the combination of Peg-IFN, GM-CSF, and HBV vaccination significantly improved HBsAg loss and seroconversion [23]. Further investigation is needed to determine the optimal combination of GM-CSF and Peg-IFN and to elucidate the immunologic mechanisms by which GM-CSF underperforms within Peg-IFN-based regimens.

Peg-IFN may induce durable off-treatment response, allowing a successful NA discontinuation. In the present study, most patients sustained serologic response after Peg-IFN cessation, and some patients achieved late HBsAg loss during follow-up. However, Peg-IFN achieved less satisfactory virological and biochemical response than NA monotherapy. After initiating Peg-IFN therapy, a high proportion of patients experienced ALT elevation, with some maintaining abnormal levels throughout treatment, while one patient had a peak ALT level exceeding 10 × ULN. During follow-up, the ALT levels return to normal range. Moreover, ALT elevation did not occur accompanied with HBV viremia. ALT flare may reflect the immune activation induced by Peg-IFN [24]. However, in the present study, early ALT elevation was not associated with HBsAg loss in the multivariate analysis.

Baseline HBsAg levels and the early dynamics during Peg-IFN treatment are considered key predictors of HBsAg loss [1]. In our post hoc analysis, baseline HBsAg level or its decline at week 12 or 24 was associated with end-of-treatment HBsAg loss and HBsAg seroconversion. Recently, novel HBV biomarkers have demonstrated potential roles in optimizing CHB management. HBcrAg and HBV RNA levels, which reflect intrahepatic cccDNA levels [25], may be associated with HBsAg loss. The present study showed that 24-week HBV RNA decline was an independent predictor of end-of-treatment HBsAg loss. It is noteworthy that the probability of HBsAg loss may vary over the course of therapy, and may not be accurately determined by certain HBV biomarkers at a single timepoint. A longitudinal model utilizing HBsAg dynamics is a reliable method for predicting HBsAg loss following Peg-IFN or long-term NA therapy [14], which may be useful in guiding treatment decisions.

One of the main drawbacks of Peg-IFN is the frequent side effects that limit its tolerability. As expected, the incidence and severity of adverse events were markedly higher during combination therapy than NA treatment. The common adverse events were those known to occur with Peg-IFN. The addition of GM-CSF to Peg-IFN could reduce the incidence of neutropenia, but may increase incidence of pyrexia, headache and rash. However, the dropout rates were comparable between EP group and EPG group. From cost-effectiveness and safety perspectives, extended-duration Peg-IFN therapy should employ baseline and early response markers to target patients most likely to achieve a functional cure and guide treatment decisions.

This study has limitations. It should be noted that our findings are applicable only to non-cirrhotic patients with viral suppression and low HBsAg titers as individuals with high HBsAg burden or cirrhosis were excluded. Moreover, the low proportion of female subjects may bias the findings regarding sex disparity in the treatment efficacy. Furthermore, an extended follow-up of this trial is essential to determine the durability of response and the long-term prognosis, including the incidence of cirrhosis and hepatocellular carcinoma. In addition, patients who relapse after treatment should be closely monitored and retreated when necessary to avoid decompensation. Finally, as novel direct-acting antivirals and immunomodulators emerge, including small interfering RNA (siRNA), antisense oligonucleotides (ASOs), checkpoint inhibitors, and toll-like receptor agonists, etc., future trials are warranted to identify optimal combination regimens that offer improved efficacy and tolerability.

In summary, among CHB patients undergoing long-term NA treatment who achieved viral suppression and had low HBsAg levels, adding Peg-IFN enhanced the rates of HBsAg loss and HBsAg seroconversion, whereas the addition of GM-CSF demonstrated no improved efficacy benefit. Age, early on-treatment HBsAg level, and 24-week HBV RNA decline correlated with HBsAg loss. The HBsAg dynamics during therapy is a strong predictor of HBsAg loss under Peg-IFN-based therapy.

## Data availability statement

Data are available upon reasonable request and with the permission of the sponsor. All requests for raw and analyzed data are promptly reviewed by Qin Ning (qning@vip.sina.com), to verify if the request is subject to any confidentiality obligations. Patient-related data not included in the paper were generated as part of clinical trials and may be subject to patient confidentiality. Any data that can be shared will be released via a data use agreement.

## Supplementary Information

Below is the link to the electronic supplementary material.

Supplementary file1 (DOCX 158 KB)

Supplementary file2 (DOCX 152 KB)

Supplementary file3 (DOC 196 KB)

## Abbreviations

AEs: Adverse events
ALT: Alanine aminotransferase
AST: Aspartate aminotransferase
AUROC: Area under the receiver operating characteristic curve
cccDNA: Covalently closed circular DNA
CHB: Chronic hepatitis B
EOF: End of follow-up
EOT: End of treatment
ETV: Entecavir
GM-CSF: Granulocyte–macrophage colony-stimulating factor
HBcrAg: Hepatitis B core-related antigen
HBeAg: Hepatitis B e antigen
HBsAg: Hepatitis B surface antigen
HBsAb: Hepatitis B surface antibody
HBV: Hepatitis B virus
LLOD: Lower limit of detection
NA: Nucleos(t)ide analog
Peg-IFN: Peginterferon alfa
ROC: Receiver operating characteristic
ULN: Upper limit of normal
