# Supplementary material for: Efficacy and safety of entecavir, peginterferon alfa-2b and GM-CSF combination therapy: the anchor randomized controlled trial
Source: Hepatol Int. 2025 Dec 9;20(1):31–45. doi: 10.1007/s12072-025-10977-2 (PMC12923447; doi:10.1007/s12072-025-10977-2)

Efficacy and safety of entecavir, peginterferon alfa-2b and GM-CSF combination therapy: The anchor randomized controlled trial

Di Wu^1^, Da Huang^1^, Shifang Peng^2^, Yongping Chen^3,4^, Fengchun Yang^1^, Xiaoyun Zhang^1^, Lei Fu^2^, Lanman Xu^5, 6^, Jiaji Jiang^7^, Qi Zheng^7^, Xinyue Chen^8^, Yali Liu^8^, Xiaoguang Dou^9^, Ke Ma^1^, Dong Xi^1^, Peng Wang^1^, Li Sun^10^, Ruoyi He^10^, Yuchen Tian^11^, Ping Yin^11^, Weiming Yan^1^*, Meifang Han^1^*, Qin Ning^1^*

| **Contents** | **Page** |
| --- | --- |
| **Method** | **3** |
| **Supplementary Fig. 1** | **4** |
| **Supplementary Fig. 2** | **4** |

**Method:**

The overall construction process of the model is as follows:

1) Two separate multilevel linear mixed models (MLMMs) are fitted for patients who failed to achieve HBsAg loss (group-0) and those who achieved HBsAg loss (group-1) before the end of the follow-up period, respectively. The parameters for these two models are denoted as $\theta_{0}$ and $\theta_{1}$. The structure of these models is as follows:

$$Log(qHBsAg) = \mu_{qHBsAg,i}+\gamma_{qHBsAg,i^{t}}$$

the parameters of the model follow the following distributions:

$\left( \begin{aligned} \mu_{\rho，i} \\ \gamma_{\rho，i} \end{aligned} \right) \sim M$*VN*$\left( \begin{aligned} \mu_{\rho} \\ \gamma_{\rho} \end{aligned} \right)\left( \begin{matrix} {\sigma^{2}}_{\mu_{\rho}} & \sigma_{\mu_{\rho},\gamma_{\rho}} \\ \sigma_{\mu_{\rho},\gamma_{\rho}} & {\sigma^{2}}_{\gamma_{\rho}} \end{matrix} \right)$

1. Both the fitting of MLMMs and the estimation of predicted values were performed using Bayesian Markov-Chain Monte-Carlo (MCMC) algorithms. $\mu_{\rho},$ $\gamma_{\rho}$, ${\sigma^{2}}_{\mu_{\rho}}$, ${\sigma^{2}}_{\gamma_{\rho}}$, $\sigma_{\mu_{\rho},\gamma_{\rho}}$ were estimated using the Bayesian MCMC algorithm when fitting the MLMM. For every Bayesian MCMC simulation, two chains were used with sufficient lengths (2000 iterations in total, with the first 1000 iterations burnt) and the convergence between two MCMC chains were assessed with visual inspections on traceplots. All Bayesian MCMC simulations used uninformative priors generated by the algorithm.
2. Then, assuming a new patient with longitudinal biomarker history up to time $t$, $y_{t}$ was available, the algorithm computed the likelihood of $y_{t}$ conditional on $\theta_{0}$ and $\theta_{1}$ respectively: that is, $P(y_{t}|\theta_{0})$and $P(y_{t}|\theta_{1})$. By Baye’s rule, the probability that the patient belonged to group-g (where g∈{0,1}) was:

$$P(G=g|y) = \frac{\omega_{g}P(y_{t}|\theta_{g})}{\sum_{h=0}^{1} P(y_{t}|\theta_{h})}$$

where ω = {$\omega_{0}$,$\omega_{1}$} was a length-2 vector denoting the prior weight of group-0 and group-1 patients.

**Supplementary Fig. 1** Comparison of FIB-4 indices and FibroScan measurements across different groups. Mean FIB-4 indices (Dots represent mean values, error bars the SEM) across different groups. Levels of significance: Blue indicates the difference between EPG group and E group: p <0.0001 at week 24, p <0.0001 at week 48, p <0.0001 at week 72, p <0.0001 at week 96, p=0.6983 at week 120; Red indicates the difference between EP group and E group: p <0.0001 at week 24, p <0.0001 at week 48, p <0.0001 at week 72, p <0.0001 at week 96, p=0.8332 at week 120 (Two-way ANOVA); Black indicates the difference between EP group and EPG group: p=0.1342 at week 24, p=0.2984 at week 48, p=0.6805 at week 72, p=0.6797 at week 96, p=0.9712 at week 120; Mean FibroScan measurements (Dots represent mean values, error bars the SEM) across different groups. Levels of significance: Blue indicates the difference between EPG group and E group: p=0.1538 at week 24, p=0.0090 at week 48, p =0.0209 at week 72, p =0.0125 at week 96, p=0.4116 at week 120; Red indicates the difference between EP group and E group: p=0.0658 at week 24, p =0.0067 at week 48, p=0.0605 at week 72, p=0.1253 at week 96, p=0.1715 at week 120; Black indicates the difference between EP group and EPG group: p=0.9251 at week 24, p=0.9933 at week 48, p=0.9116 at week 72, p=0.6100 at week 96, p=0.8544 at week 120 (Two-way ANOVA).


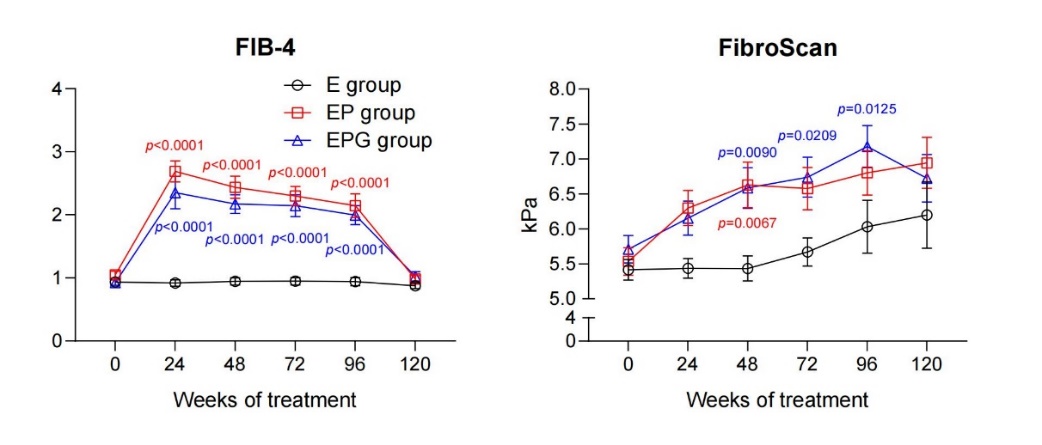


**Supplementary Fig.2** The confusion matrix evaluating the performance of the model in predicting HBsAg loss at week 96. (A) The confusion matrix evaluating the performance of the model in predicting HBsAg loss at week 96 in EP group. (B) The confusion matrix evaluating the performance of the model in predicting HBsAg loss at week 96 in EPG group.

A


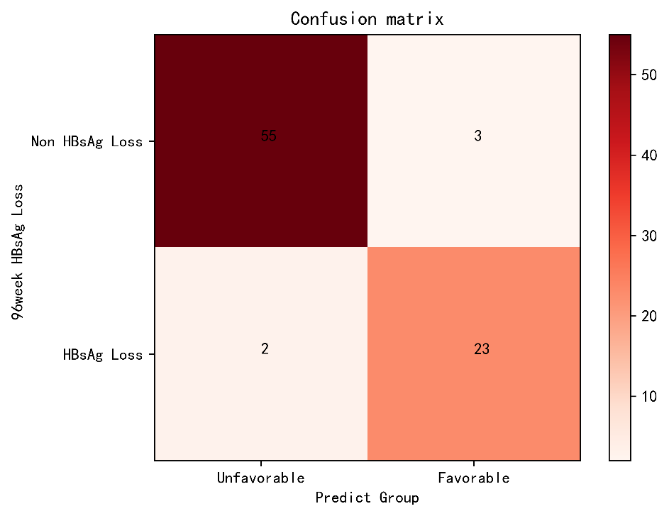


B


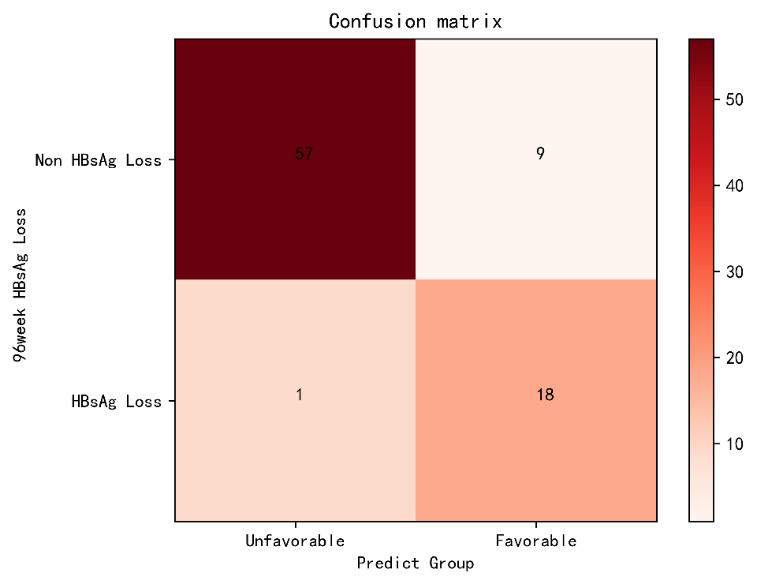

Supplement: Supplementary file 1 — Supplementary file1 (DOCX 158 KB) [file 12072_2025_10977_MOESM1_ESM.docx]
