# Supplementary material for: Efficacy and safety of entecavir, peginterferon alfa-2b and GM-CSF combination therapy: the anchor randomized controlled trial
Source: Hepatol Int. 2025 Dec 9;20(1):31–45. doi: 10.1007/s12072-025-10977-2 (PMC12923447; doi:10.1007/s12072-025-10977-2)
Supplement: Supplementary file 2 — Supplementary file2 (DOCX 152 KB) [file 12072_2025_10977_MOESM2_ESM.docx]

**CLINICAL STUDY PROTOCOL**

**Combination therapy of pegylated interferon-alpha-2b, nucleoside/nucleotide analogues (NAs) and GMCSF in NA-experienced chronic hepatitis B patients: A prospective, randomized, open-label trial (Anchor study)**

Sponsor The Chinese National Twelfth Five Years Project

in Science and Technology (2013ZX10002003),

The Chinese National Thirteenth Five Years

Project in Science and Technology

(2017ZX10202201)

Principal investigator Prof. Ning Qin

Principal site Tongji Hospital of Tongji Medical College, Hua

Zhong University of Science and Technology

Type Protocol

Version 1.3

Date Nov 19, 2015

Confidentiality Statement

This information contained in this document, especially unpublished data, is the property of Tongji

Hospital of Tongji Medical College, Hua Zhong University of Science and Technology (or under its control), and therefore provided to you in confidence as an investigator, potential investigator or consultant, for review by you, your stuff and an applicable Institutional Review Committee.

| Protocol Title | Combination therapy of pegylated interferon-alpha-2b, nucleoside/nucleotide analogues (NAs) and GMCSF in NA-experienced chronic hepatitis B patients: A prospective, randomized, open-label trial (Anchor study) |
| --- | --- |
| Number of Centers: | 6 |
| List of study centers: | 1. Department and Institute of Infectious Disease, Tongji Hospital,   Tongji Medical College, Huazhong University of Science and  Technology, Wuhan, China   1. Department of Infectious Disease, The First Affiliated Hospital of   Wenzhou Medical University, Wenzhou, China   1. Beijing You'an Hospital, Capital Medical University, Beijing, China 4. Liver Research Center, First Affiliated Hospital of Fujian Medical   University, Fuzhou, China   1. Department of Infectious Diseases, Xiangya Hospital, Central   South University, Changsha, China   1. Department of Infectious Disease, Shengjing Hospital of China   Medical University, Shenyang, China |
| Principal investigator: | Qin Ning |
| Principal site: | Department and Institute of Infectious Disease, Tongji Hospital, Tongji  Medical College, Huazhong University of Science and Technology,  Wuhan, China |
| Address | No. 1095, Jiefang Avenue, Wuhan, China. 430030 |
| Tel/Fax: | +86 278366 2391 |
| E-mail: | qning@vip.sina.com |
| Independent Ethics Committee: | the independent central ethics committee of Tongji Medical College at  Wuhan |
| Co-Investigator: | Yongping Chen, Xinyue Chen, Jiaji Jiang, Deming Tan, Xiaoguang Dou, Xiaoping Luo |

SYNOPSIS OF PROTOCOL

| TITLE | Combination therapy of pegylated interferon-alpha-2b, nucleoside/nucleotide analogues (NAs) and GMCSF in NA-experienced chronic hepatitis B patients: A prospective, randomized open-label trial (Anchor study) |
| --- | --- |
| SPONSOR | The Chinese National Thirteenth Five Years Project in Science and Technology (2017ZX10202201) |
| INDICATION | NA-experienced CHB |
| OBJECTIVES | Primary: To compare the efficacy (HBsAg loss) and safety of pegylated interferon alfa-2b therapy plus entecavir (ETV) with or without GMCSF or ETV alone in NA-experienced CHB patients who had sustained HBV DNA suppression by ETV treatment. |
| TIRAL DESIGN | A multi-center, randomized, prospective, open-label Phase IV clinical trial. Patients will be randomized 1:1:1 in the IFN+ETV +GMCSF combination therapy group, IFN+ETV group or ETV group. |
| NUMBER OF PATIENTS | Total N 257 |
| TARGET POPULATION | The subjects who pre-treated by ETV for at least 1 year and achieved HBV DNA ≤10^3^ copies/ml & HBsAg <3000 IU/ml. Patients were excluded if they had previously developed drug resistance to ETV, or received IFN or systemic antiviral therapy within the previous 6 months. Patients were also excluded if they were coinfected with the human immunodeficiency virus, hepatitis C virus or hepatitis D virus or if they had decompensated liver disease (defined as a Child-Pugh Score B or C), pregnancy or lactation, or any other contraindication for IFN therapy |
| LENGTH OF STUDY | 96 weeks treatment  24 weeks follow up |
| DURATION OF STUDY | 42 months after first patient randomized |
| INVESTIGATIONAL PRODUCTS  DOSE/ROUTE/REGIMEN | Group 1: Entecavir 0.5mg once per day; P.O.  Group 2: Entecavir 0.5mg once per day (cessation at week 48); P.O. Pegylated interferon alfa-2b 180ug once per week; S.C.  Group 3: Entecavir 0.5mg once per day (cessation at week 48); P.O. Pegylated interferon alfa-2b 180ug per week; S.C. plus GM-CSF (Teerli, Xiamen Amoytop Biotech Co., Ltd, China) 75 μg once-daily for the first 5 consecutive days each month; S.C. |
| ASSESSMENT OF EFFICACY | Primary Endpoint:  HBsAg loss at Week 96.  Secondary Endpoint:   1. HBsAg seroconversion 2. Proportion of patients who achieve undetectable HBV DNA 3. Quantitative HBsAg measurement 4. HBeAg seroconversion 5. ALT normalization 6. Sustained virologic response at the end of follow-up |
|  | Histology improvement (option) |
| SAFTY: | Adverse events, vital signs and clinical laboratory parameters. |
| PROCEDURES (SUMMARY) | After signing informed consent and meeting screening parameters, patients are randomized to 1 of 3 treatment groups. patients will be evaluated at Day 1, Week 2, 4, 8, 12, 16, 20, 24, 28, 32, 36, 40, 44, 48, 52, 56, 60, 64, 68, 72, 76, 80, 84, 88, 92, 96, 108 and 120. |
| STATISTICAL ANALYSES:  Population: | Intent-to-treat (patients who receive at least one dose of test drug)  Per-protocol (patients who follow the study protocol and complete the study)  All patients receiving at least one dose of study medication and have a subsequent safety assessment will be considered for analyses of safety. |
| Efficacy: | The descriptive statistics depending on the type of variables will be as follows:  Quantitative variable: size, mean, standard deviation, minimum and maximum.  Qualitative, ordinal variables: frequency and percentage per class.  The percentage and 95% CI are employed to serve the calculation of response rates for the primary endpoint and secondary endpoints. The Chi-square test is used for bivariate variables. |
| Safety: | Adverse events   - Adverse events will be assigned preferred terms and categorized into body systems according to the Medical Dictionary for Drug Regulatory Affairs (MEDDRA) classification of the WHO terminology.   Laboratory Safety Data   - The laboratory data will be analyzed according to the   International Conference on Harmonization (ICH) Guideline for Clinical Safety Data Management: Definitions and Standards for  Expedited Reporting (E2). |

**TABLE OF CONTENTS**

GLOSSARY OF ABBREVIATIONS…………………………………..………………………………………..…..8

1. BACKGROUD AND RATIONALE…………………………………….……………………………….…..…9
2. INVESTIGATIONAL PRODUCTS…………………………………….……………………………….……12
   1. Peg-IFN-α-2b…………………………………….………………………………………..……….12
   2. ETV…………………………………………………………………………………………...…….12
   3. rhGM-CSF……………………………………………………………….………….13
3. OBJECTIVES OF STUDY…………………………………………………….………………………….….13
4. STUDY DESIGN……………………………………………………………….………………………….….13
   1. Overview of study design……………………………………...………………..…………….….13
   2. Numbers of patients………………………….…………………...……………..………….…….14
   3. Assignment to treatment groups…………….………..…………..……………………….…….14
   4. Dosing Regimen…………….………..………………………………....…………………..…….14
   5. Centers……..…………………………………………………………....…………………..…….14
5. ENDPOINTS………………………………………………..………………………………………..……….15
   1. Efficacy…………………………………………….…..……………………………………..…….15
      1. Primary endpoint…………………………………………………..…………...…….15
      2. Secondary endpoint………….…………………..……………..………………..….15
   2. Safety………………………………………………………..……………..………………...…….15
6. TARGET POPULATION…………………………………………..……………..……………………….….15
   1. Overview…………………………………………….………..……………..……………………..15
   2. Recruitment procedures………………………….………..…………..…..……………….…….15
   3. Inclusion criteria…………………………………….………..……………..……………….…….16
   4. Exclusion criteria………………………………….………..……………....……………...…..….16
7. SCHEDULE OF ASSESSMENTS AND PROCEDURE……..……………....……………..…………….18
   1. Schedule of assessments……………………….………..………………....………………..….18
   2. Screening examination and eligibility screening form…..……...………....……………..……20
   3. Procedures for enrollment of eligible subjects….………..…………...…....….………..….….21
   4. Clinical assessments and procedures………….………..…………...…....………………..….22
      1. Efficacy………………………………….………..…..……....…………………...….22
      2. Safety…………………..……………….………..……...…....…………………...….22
   5. Laboratory assessments……..……………….………..……………....……...……………..….22
   6. Subject medication diary review…………….………..……………....………...……………….23
   7. Concomitant medication and treatment…….………..……………....………...…………...….23
   8. Criteria for premature withdrawal…………….………..……………....…………...………..….24
8. SCHEDULE OF INVESTIGATION MEDICAL PRODUCT..……………....…………………………..….24
   1. Stopping rules………………..……………….………..……………....…...……..…………..….24
   2. Missing Consecutive Doses..……………….………..……………....…………..…………..….25
   3. Accountability of study medicinal product….………..……………....…………..………….….25
   4. Assessment of Compliance..……………….………..……………....…………..…………..….25
   5. Destruction of the study medicine………….………..……………....…………..…...……..….26
9. SAFETY INSTRUCTION AND GUIDANCE…….………..……………....………………………...….….26
   1. Insurance and indemnity…………….………..……………....………..….……………....…….26
   2. Adverse events and laboratory abnormalities……………....………..….……………....…….26
      1. Clinical adverse events………...………..….……………………………………....26
      2. Intensity…………....………..……………………………………..……………..…..26
      3. Drug-Adverse Event Relationship……...………………………..…………….…...27
      4. Serious Adverse Events…..……………………………………..……………...…..27
      5. Treatment and Follow-up of Adverse Events……………..………..……………..28
      6. Laboratory Test Abnormalities…………………………………..…………………..28
      7. Follow-up of Abnormal Laboratory Test Values……………………………….…..28

9.3 Handling of safety parameters…..….…………………...………………………………...……..29

- - 1. Reporting of Adverse Events….……………………..………..…………….………..29
    2. Reporting of Serious Adverse Events………………………………..…………….29
    3. Pregnancy………………………………………………..……..…………………….29
    4. Warnings and precautions……………………………………..……..……………..30

1. THE COMPLETE STATISTICAL ANALYSIS PLAN…………………………….………………………...30
   1. Primary and secondary study endpoints…………………………..……..…………...………..30
      1. Primary endpoints………………………………..……..…………………………....30
      2. Secondary endpoints……………………………..……..………………………......30
      3. Safety………………………………..……………..……..…………………………..30
   2. Statistical methods………………………..……………..……..…………...…………………….31
      1. Primary Variables……………………..…………..……..…………………………..31
      2. Secondary Variables…………………..……………..……..…….………………....31
      3. Sample Size……………………..……..……………..……..…….………………....31
      4. Analysis populations……………..……..……………..……..……………….……..31
      5. Efficacy Analysis……………..……..……………..……..…………..….…………..31
      6. Exclusion of Data from Analysis…..…………...……..…………..………………..32
         1. Intent-to-Treat Population and modified ITT Population…..………...32
         2. Safety Data Analysis..……..……………..……..……..………………..32
2. DATA COLLECTION, MANAGEMENT, AND ASSURANCE……..…………..…………………...……..32

# GLOSSARY OF ABBREVIATIONS

| *AE* | *Adverse event* |
| --- | --- |
| *AFP* | *Alpha-fetoprotein* |
| *ALT* | *Alanine aminotransferase* |
| *ANA* | *Anti-nuclear antibody* |
| *AST* | *Aspartate aminotransferase* |
| *BUN* | *Blood urea nitrogen* |
| *CHB* | *Chronic hepatitis B* |
| *CRF* | *Case report form* |
| *DNA* | *Deoxyribonucleic acid* |
| *ECG* | *Electrocardiogram* |
| *EOT* | *End of treatment* |
| *ESF* | *Eligibility screening form* |
| *ETV* | *Entecavir* |
| *HAV* | *Hepatitis A virus* |
| *HBcAg* | *Hepatitis B core antigen* |
| *HBeAb* | *Hepatitis B e antibody* |
| *HBeAg* | *Hepatitis B e antigen* |
| *HBsAb* | *Hepatitis B s antibody* |
| *HBsAg* | *Hepatitis B s antigen* |
| *HBV* | *Hepatitis B virus* |
| *HCG* | *Human choionic gonadotophin* |
| *HCV* | *Hepatitis C virus* |
| *HDV* | *Hepatitis D virus* |
| *HIV* | *Human immunodeficiency virus* |
| *ICH* | *International Conference on Harmonization* |
| *IEC* | *Independent ethics committee* |
| *IFN* | *Interferon* |
| *IgM* | *Immunoglobulin M* |
| *IRB* | *Institutional review board* |
| *ITT* | *Intention-to-treat* |
| *ml* | *Milliliter* |
| *NA* | *Nucleos(t)ide analogue* |
| *PEG-IFN* | *Pegylated Interferon* |
| *SAE* | *Serious adverse event* |
| *T3* | *triiodothyronine* |
| *T4* | *Thyroxin* |
| *TSH* | *Thyroid stimulating hormone* |
| *ULN* | *Upper limit of normal* |
|  |  |

**Study Design and Conduct**

# Background and Rationale

Hepatitis B virus (HBV) infection continues to be a major public health burden in our country. Hepatitis B surface antigen (HBsAg) loss is rarely achieved under exiting antiviral regimens. Due to the hepatophilic property and high variability of HBV, and more importantly, the HBV genome forms a stable minichromosome, namely covalently closed circular DNA (cccDNA), in the nuclei of infected hepatocytes, which is difficult to eliminate completely, together with host immune tolerance or immune deficiencies, enabling HBV to persist its infection and contributing to liver fibrosis, cirrhosis, even liver cancer eventually.

Exiting antiviral regimens and optimized therapy can inhibit viral replication and cccDNA production to some extent, but its unlikely to eliminate cccDNA in nucleus completely. It accounted for the suboptimal treatment response and the relapse after therapy discontinuation. If the intrahepatic cccDNA cannot be cleared, amounts of CHB patients will still face the risk of recurrence and lead to treatment failure, even if they had achieved HBV DNA suppression and HBeAg seroclearance by previous antiviral drugs.

Unlike HBeAg seroclearance, HBsAg loss represents a durable immunologic control over the virus and complete suppression of HBV replication, which is considered as a “functional cure” for CHB. It always occurs after the reduction of HBsAg, and the decline in quantitative HBsAg (qHBsAg) often indicates the efficacy of antiviral therapy. Previous study revealed that serum HBsAg correlated with intrahepatic cccDNA for HBeAg-positive CHB patients. The reduction of qHBsAg indicated the decline in cccDNA during IFN treatment and predicted the sustained off-treatment response. A clinical trial combinational use of Peg-IFN and adefovir dipivoxil suggested that combination therapy could reduce serum viral load and nuclear cccDNA, and correlated with the reduction of qHBsAg. For HBeAg-negative CHB patients with genotype D, the decline in qHBsAg or HBV DNA <2 log at week 12 under IFN treatment predicted the poor treatment efficacy, and the discontinuation of IFN should take into consideration.

IFN or NAs mono-therapy cannot exert satisfied antiviral effect on CHB patients. Its urgent to take their advantages and disadvantages to improve treatment efficacy and reduce therapy cost. Existing domestic antiviral strategies are mainly based on foreign guidelines, and most clinical research data are limited to mono-therapy. Therefore, new treatment approaches such as optimal combination therapy with the approved antivirals or emerging novel therapeutics integrated viral clearance and immune modulation are needed to establish the Chinese special antiviral strategy toward the functional cure of CHB.

Different characteristics, mechanisms of action and antiviral activities of NAs and IFN provide the possibility of combining these two types of agents for breaking immune tolerance and improving antiviral efficacy for CHB. Our previous prospective, randomized controlled trial (OSST study) which published in Journal of Hepatology reported that switching to 48-week course of Peg-IFN in HBeAg-positive CHB patients who achieved virologic remission after long-term ETV treatment led to significantly increased rates of HBeAg seroconversion (14.9%) and HBsAg loss (8.5%). It received extensive attention from international and domestic liver disease experts and the author was invited to publish a special letter on antiviral treatment strategies for NA-treated patients in the Journal of Hepatology.

Several studies have demonstrated that Peg-IFN combined with lamivudine or adefovir dipivoxil for 96 weeks can obtain higher rates of HBeAg seroconversion and HBsAg seroconversion than the standard duration of 48 weeks, and their safety are consistent. It is suggested that combination therapy of Peg-IFN and NAs are well tolerant and tend to achieve better antiviral treatment efficacy. However, more large-scale multi-center clinical research are needed to further confirm.

IFN and Peg-IFN exert immune-mediated anti-HBV effect with no drug resistance, patients with undetectable HBV DNA at the end of IFN treatment have the opportunity to obtain a durable virological response even HBsAg loss. Therefore, in order to avoid the risk of resistance in long-term antiviral treatment, the guidelines for preventing and treating CHB in various countries recommend Peg-IFN as the preferred regimes, and further indicated that granulocyte colony stimulating factor (G-CSF) or granulocyte-macrophage colony stimulating factor (GM-CSF) may be taken into consideration for patients with notable neutropenia.

Recombinant human granulocyte-macrophage stimulating factor (rhGM-CSF) is a genetically recombinant protein that can promote the proliferation and differentiation of hematopoietic progenitor cells, induce granulocytes and monocytes/macrophages to mature and release into peripheral blood, and enhance their activity. In addition, it promotes dendritic cells, endothelial cells, keratinocytes and many other cells. Therefore, it widely used to prevent and treat leukopenia caused by tumor radiotherapy and chemotherapy. Combined with IFN can reduce and reverse the development of leukopenia. What’s more, it can activate the functions of immune cells including monocytes and macrophages to improve the antiviral efficacy.

A study randomizely assigned CHB patients to receive IFN and GM-CSF, or IFN and lamivudine for 15 months. Compared with IFN and lamivudine therapy, combination of IFN and GM-CSF treatment exhibited a significantly higher sustained virological response rate (40% vs 28%) at the end of treatment, suggesting the enhancement effect of GM-CSF on IFN treatment for CHB patients. Furthermore, there are many reports in the literature that GM-CSF can be used as an immune adjuvant to enhance the response of hepatitis B vaccine.

GM-CSF has the following applications in the treatment of hepatitis: (1) to treat CHB combined with IFN; (2) to treat chronic hepatitis C; (3) to be used as an adjuvant for hepatitis B vaccine; (4) to be used in hepatitis patients undergone IFN therapy with bone marrow suppression. It is safe and effective to obtain viral clearance when combined with IFN and GM-CSF. About 70% to 90% of patients may experience a decrease in total white blood cells, neutrophils, and platelet counts under IFN therapy. Among them, the decrease in neutrophil counts is the most common. The doses of IFN need to be adjusted when the decrease reaches a certain level. GM-CSF can increase the levels of white blood cells and neutrophils to help maintain the effective doses and durations of IFN, thereby improving patients’ compliance, so it is widely used in patients with hepatitis B and C during IFN treatment.

However, the combination antiviral therapy of IFN and GM-CSF is limited to clinical experience and small sample reports. How to standardize the treatment, what indicators should be used to optimize clinical practice, or how to enhance the effect of IFN? There remains a lack of large-sample multi-center standardized clinical data.

Professor Wang Guiqiang from the First Hospital of Peking University designed the Y-type PEGylated recombinant human IFN alpha 2b combined with GM-CSF therapy for HBeAg-positive CHB in a multi-center, randomized, parallel controlled clinical trial in 2014. Using recombinant human Peg-IFN-α-2b (Pegabine®) as the parallel control, they preliminarily explored the treatment efficacy and safety of Peg-IFN combined with GM-CSF in HBeAg-positive CHB patients and compared the medication compliance of Peg-IFN in the two treatment plans as well, to provide a basis for standardizing the combinational therapy. The project has been reviewed by the ethics committee and multi-center clinical study is being carried out nationwide. The main target population is naïve CHB patients with HBeAg positivity, while NA-treated patients are excluded from the project.

Focusing on increasing the rate of HBsAg loss, the central goal of national "Twelfth Five-Year" viral hepatitis plan, our study proposes a multi-target combination intervention to improve HBsAg loss rate for CHB patients who had received long-term NAs treatment but not achieved HBeAg seroconversion. Based on the data from previous OSST study, the sequential combination therapy of NAs and IFN are designed, and the immunomodulatory factor GM-CSF is added timely. It is planned to establish a cohort in multiple centers and fulfill the new treatment strategy.

This study intends to adopt a prospective, multi-center, randomized, controlled, open-labeled trial design. The enrolled CHB patients will be assigned to three treatment groups according to the principle of random (1:1:1). Patients in Group I are treated with ETV for 96 weeks; patients in Group II are treated with Peg-IFN for 96 weeks, combined with ETV during the first 48-week; Group III are treated with Peg-IFN for 96 weeks, combined with ETV and GM-CSF during the first 48-week. All patients will be followed-up for 24 weeks after treatment. It is expected that the HBsAg loss rate in Group II and III will increase by more than 10% compared with Group I after treatment, which indicates a better clinical outcome. The success of this project will improve the treatment efficacy of existing antiviral therapy, and enable CHB patients to safely stopping NAs in advance to avoid drug resistance. More importantly, amounts of CHB patients with suboptimal treatment efficacy will obtain HBeAg seroconversion even HBsAg seroconversion, which can reduce the occurrence of liver cirrhosis and liver cancer. It will produce huge social and economic benefits.

# Investigational Products

## 2.1 Peg-IFN-α-2b

IFN is a cytokine produced by lymphocytes and can be divided into types α, β and γ according to it‘s structure. IFN-α has the strongest antiviral effect. Recombinant IFN-α approved for anti-HBV treatment included IFNα-1b, IFNα-2b, IFNα-1a, IFNα-2a and IFNαcon-1, etc. in our country. IFN-α can exert dual functions of anti-virus and immune regulation. It triggers the activation of intracellular enzymes and produces antiviral proteins including 2', 5'oligoadenylate synthase (2', 5'-AS), phosphodiesterase and protein kinase, when IFN-α binds to specific receptors on target cells. 2',5'-AS can degrade viral mRNA through activating intracellular nuclease. Phosphodiesterase can degrade the tail of viral tRNA. Protein kinase can block the initiation of viral protein synthesis to inhibit viral replication. IFN-α can also enhance the expression of HLA-I antigens on the cell membrane to promote CTL to recognize and attack target cells, the activity of NK cells and the killing ability of macrophages against virus-infected cells. In addition, IFN can regulate the production of IL-1, IL-2 and tumor necrosis factor (TNF) generated from Th2 cells to help immune system clear virus. Peg-IFN, which has long-acting antiviral effect and is well tolerant, has been used as a routine drug for the antiviral treatment of CHB at present.

## 2.2 ETV

ETV is an analogue of epoxy hydroxycarbodeoxyguanosine, which has a potent and selective anti-HBV effect with no inhibition on human mitochondrial gamma polymerase. It can interfere with the courses of initiation, reverse transcription and DNA synthesis of HBV replication. Similar to other nucleoside analogs, ETV needs to enter the cell to be phosphorylated to produce an active substance (triphosphate entecavir), its intracellular half-life is about 15 hours. Drug sensitivity test using HBV transfected cells in vitro showed that ETV has the strongest antiviral activity, which is more than 300 times higher than other nucleoside analogs. A randomized double-blind controlled clinical trial suggested that for HBeAg-positive CHB patients, the rates of HBV DNA<300 copies/mL, ALT normalization and liver histology improvement were 67%, 68% and 72% after 48-week ETV treatment, which significantly higher than those receiving lamivudine. However, the HBeAg seroconversion rate was similar in the two groups (21% and 18%). For HBeAg-negative patients, the rates of undetectable HBV DNA, ALT normalization and liver histology improvement were 90%, 78% and 70% after 48-week ETV treatment. A Japanese study showed that the 3-year cumulative resistance rate of entecavir was 1.7% to 3.3%.

## 2.3 rhGM-CSF

Recombinant human GM-CSF is a non-glycosylated acidic protein produced by recombinant genetic engineering technology. It has only 1 subunit consist of 127 amino acids with a relative molecular weight of 14455. It is mainly used to prevent and treat leukopenia caused by chemotherapy or radiotherapy. It can promote the proliferation and differentiation of hematopoietic progenitor cells, the maturation and release of granulocytes and mononuclear macrophages, etc. More importantly, it helps to reduce the occurrence of leukopenia during IFN treatment in this study.

# 3. Objectives of study

To compare the efficacy and safety of Peg-IFN therapy in combination with ETV with or without GM-CSF in NA-suppressed CHB patients who had HBV DNA<1000 copies/ml and HBsAg<3000 IU/ml.

# 4. Study design

## Overview of study design

This study is a multi-center, randomized, prospective, open-label Phase IV clinical trial (Fig 1), CHB patients who had HBV DNA<1000 copies/ml and HBsAg<3000 IU/ml by NAs treatment, were randomized at a ratio of 1:1:1 to one of 3 groups as follows:

Arm A: ETV 0.5mg once daily for 96 weeks

Arm B: Peg-IFN-α-2b 180 ug/week S.C. for 96 weeks plus ETV 0.5mg once daily from day 1 to 48 weeks

Arm C: Peg-IFN-α-2b 180 ug/week S.C. for 96 weeks plus ETV 0.5mg once daily from day 1 to 48 weeks plus GM-CSF from day 1 to 48 weeks


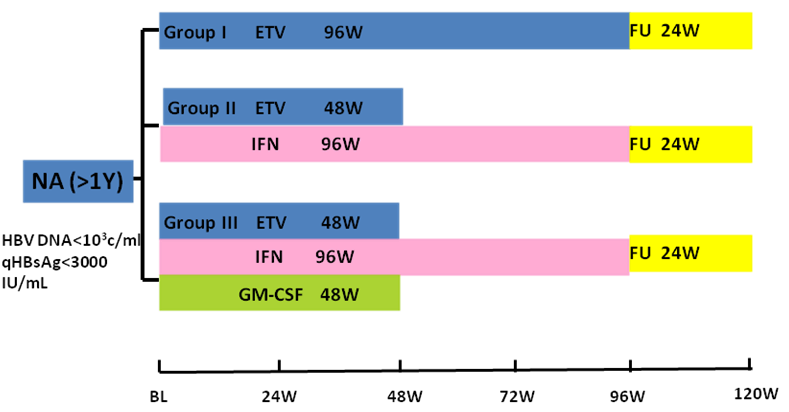


Fig 1. Study design

## Numbers of Patients

It was assumed that the HBsAg loss rate would be 10-20% in the IFN arm based on OSST study in ETV-suppressed patients, and that HBsAg loss rate in ETV arm would be 0-3%. But there is a lack of data regarding HBsAg loss rate by the novel treatment strategy using IFN in combination with GM-CSF. As a pilot and a proof of concept study, the sample size was set at approximate 80-100 per group to investigate whether this combination therapy could induce higher HBsAg loss. The total number of patients will be approximately 240-300.

## Assignment to treatment groups

All the eligible patients were randomly assigned in a 1:1:1 to one of three groups. We used interactive web response system (IWRS) for computer-generated randomisation sequences with a predefined stratification scheme. Randomisation was stratified by HBeAg (positive or negative) state and gender (male or female) at enrolment. A patient will be enrolled in the study and assigned a patient number after all protocol eligibility requirements have been met. The investigator will use the appropriate patient’s Case Report Form (CRF) and record the patient’s allocated treatment or treatment number in the CRF. It is the investigator’s responsibility to ensure that each patient receives the assigned treatment.

## Dosing Regimen

The dose of Peg-IFN-α-2b will be 180ug, administered S.C. once per week, for 96 weeks in group II and group III. The dose of ETV will be 0.5mg P.O. once per day, for 96 weeks in group I, for 48 weeks in group II and group III. GM-CSF will be administered S.C. a 5-day treatment of GM-CSF every 4 weeks, starting from the 1st day of the 1st week, 75ug once per day, periodically until 48 weeks in group III.

## Centers

This study will be conducted in 6 centers in China. These centers ere qualified by training and experience in the management of patients with HBV. Each center in expected to contribute at least 40-50 patients over the course of approximately 12 months.

Recruitment will be competitive.

List of study centers:

1. Department and Institute of Infectious Disease, Tongji Hospital, Tongji Medical College, Huazhong

University of Science and Technology, Wuhan, China

1. Department of Infectious Disease, The First Affiliated Hospital of Wenzhou Medical University,

Wenzhou, China

1. Beijing You'an Hospital, Capital Medical University, Beijing, China
2. Liver Research Center, First Affiliated Hospital of Fujian Medical University, Fuzhou, China
3. Department of Infectious Diseases, Xiangya Hospital, Central South University, Changsha, China
4. Department of Infectious Disease, Shengjing Hospital of China Medical University, Shenyang, China

# End points

## Efficacy

### Primary Endpoint

The primary efficacy parameter is HBsAg loss as measured by the Roche Elecsys HBsAg

II Quant assay (Roche Diagnostics, Penzberg, Germany; dynamic range 0.05-52,000

IU/ml), with both taken at end of treatment and 24 weeks after the end of treatment.

### Secondary Endpoint

The secondary efficacy parameters include (at end of treatment and 24 weeks after the end of treatment)

1. HBsAg positivity and seroconversion
2. Proportion of patients who achieve undetectable HBV DNA
3. Quantitative HBsAg and it’s decline
4. HBeAg seroconversion
5. ALT normalization

6. Sustained virologic response at the end of follow-up

Histology improvement (option)

## Safety

Adverse Events, vital signs and clinical laboratory parameters were assessed by investigators according to the International Conference on Harmonization (ICH) Guideline for Clinical Safety Data Management: Definitions and Standards for Expedited Reporting (E2).

- AEs (including neurological and psychiatric events), serious and non-serious, and non-serious AEs of special interest
- Laboratory test results (including thyroid function)
- Vital signs (blood pressure, heart rate, temperature)
- Growth: weight and height

# Target population

Under no circumstances are subjects who enroll in this study permitted to be rerandomized to this study and enrolled for a second course of treatment.

## Overview

NA-suppressed CHB patients who had been treated with NAs for at least one year, with HBV DNA<1000 copies/ml and HBsAg<3000 IU/ml. Subjects coinfected with HCV, hepatitis D virus (HDV), human immunodeficiency virus (HIV) or who have received IFN therapy for hepatitis B in the prior 6 months or who have liver cirrhosis or de-compensated liver disease will be excluded.

## Recruitment Procedures

Subjects will be identified for potential recruitment using pre-screening enrollment logs, Independent Ethics Committee (IEC)/Institutional Review Board (IRB)-approved newspaper/radio advertisements, and/or mailing lists prior to consenting/assenting to take place in this study.

## Inclusion Criteria

1. Male and female patients aged 18 to 65 years old at baseline;
2. Subjects who experienced ETV treatment for at least 1 year;
3. Serum HBV DNA≤1000 copies/ml;
4. HBsAg positive;
5.
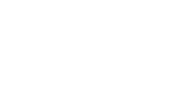

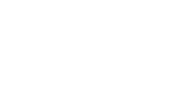
ALT >2 ULN but <10 ULN and HBeAg positive before NAs treatment;
6. Negative urine or serum pregnancy test (for women of childbearing potential) documented within the 24-hour period prior to the first dose of test drug;
7. Absence of cirrhosis determined by liver ultrasound;
8. Agree to participate in the study and sign the patient informed consent.

## Exclusion Criteria

1. Patients who had previously developed drug resistance to ETV;
2. Other antiviral, anti-neoplastic or immunomodulatory treatment (including supraphysiologic doses of steroids and radiation) *6 months prior to the first dose of randomized treatment (except for 7 days of acyclovir for herpetic lesions more than 1 month prior to first administration of randomized treatment). Patients who are expected to need systemic antiviral therapy other than that provided by the study at any time during their participation are also excluded;
3. Women with ongoing pregnancy or breast-feeding;
4. Co-infection with active hepatitis A, hepatitis C, hepatitis D (Those hospitals which have the ability to do the test will do) and/or human immunodeficiency virus (HIV);
5. Evidence of decompensated liver disease (Child-Pugh score>5). Child-Pugh>5 means, if one of the following 5 conditions are met, the patient must be excluded:
6. Serum albumin <3.5 g/L;
7. Prothrombin time ≥3 seconds prolonged;
8. Serum bilirubin >34 µmol/L;
9. History of encephalopathy;
10. History of variceal bleeding;
11. Ascites
12. History or other evidence of a medical condition associated with chronic liver disease other than viral hepatitis (e.g., hemochromatosis, autoimmune hepatitis, metabolic liver disease, alcoholic liver disease, toxin exposures, thalassemia);
13.
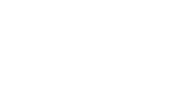
Signs or symptoms of hepatocellular carcinoma, patients with a value of alphafetoprotein >100 ng/mL are excluded, unless stability (less than 10% increase) has been documented over at least the previous 3 months. Patients with alphafetoprotein values >20 ng/mL but
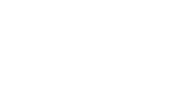
 ≤100 ng/mL may be enrolled, if hepatic neoplasia has been excluded by liver imaging;
14. Neutrophil count <1500 cells/mm^3^ or platelet count <90,000 cells/mm^3^ at screening;
15. Hemoglobin <11.5 g/dL for females and <12.5 g/dL for men;
16. Serum creatinine level >1.5 ULN in screening period.
17. Phosphorus <0.65 mmol/L;
18. ANA>1:100;
19. History of severe psychiatric disease, especially depression. Severe psychiatric disease is defined as treatment with an antidepressant medication or a major tranquilizer at therapeutic doses for major depression or psychosis, respectively, for at least 3 months at any previous time or any history of the following: a suicidal attempt hospitalization for psychiatric disease, or a period of disability due to a psychiatric disease;
20. History of a severe seizure disorder or current anticonvulsant use;
21. History of immunologically mediated disease, (e.g., inflammatory bowel disease, idiopathic thrombocytopenic purpura, lupus erythematosus, autoimmune hemolytic anemia, scleroderma, rheumatoid arthritis etc.);
22. History of chronic pulmonary disease associated with functional limitation;
23. History of severe cardiac disease (e.g., NYHA Functional Class III or IV, myocardial infarction within 6 months, ventricular tachyarrhythmias requiring ongoing treatment, unstable angina or other significant cardiovascular diseases);
24. Major organ transplantation or other evidence of severe illness, malignancy, or any other conditions, which would make the patient, in the opinion of the investigator, unsuitable for the study;
25. History of thyroid disease poorly controlled on prescribed medications, elevated thyroid stimulating hormone (TSH) concentrations with elevation of antibodies to thyroid peroxidase and any clinical manifestations of thyroid disease;
26. Evidence of severe retinopathy or clinically relevant ophthalmologic disorder (e.g. due to hypertension or diabetes mellitus, CMV retinitis, macular degeneration);
27. Patients consuming alcohol more than 20g/day for women and 30g/day for men in the 6 months preceding enrollment;
28. Evidence of drug abuse or treatment with methadone within one year of study entry.
29. Patients included in another trial or having been given investigational drugs within 12 weeks prior to screening;
30. Inability or unwillingness to provide informed consent or abide by the requirements of the study.

# Schedule of assessments and procedure

## Schedule of assessments

Table 1 Schedule of assessments

| Flowchart | Screening | Treatment period | | | | | | | | | | | | | | | | | | | | | | | | | | Follow-up | |
| --- | --- | --- | --- | --- | --- | --- | --- | --- | --- | --- | --- | --- | --- | --- | --- | --- | --- | --- | --- | --- | --- | --- | --- | --- | --- | --- | --- | --- | --- |
| Visit | V1 | V2 | V3 | V4 | V5 | V6 | V7 | V8 | V9 | V1  0 | V1  1 | V1  2 | V1  3 | V1  4 | V1  5 | V1  6 | V1  7 | V1  8 | V1  9 | V2  0 | V2  1 | V2  2 | V2  3 | V2  4 | V2  5 | V2  6 | V2  7 | V28 | V29 |
| Study  Weeks  (w) | -8w-0d | 0d | 2w  ±  3d | 4w  ±  3d | 8w  ±  3d | 12 w  ±  3d | 16 w  ±  3d | 20 w  ±  3d | 24 w  ±  3d | 28 w  ±  3d | 32 w  ±  3d | 36 w  ±  3d | 40 w  ±  3d | 44 w  ±  3d | 48 w  ±  3d | 52 w  ±  3d | 56 w  ±  3d | 60 w  ±  3d | 64 w  ±  3d | 68 w  ±  3d | 72 w  ±  3d | 76 w  ±  3d | 80 w  ±  3d | 84 w  ±  3d | 88 w  ±  3d | 92 w  ±  3d | 96 w  ±  3d | 108 w  ±  7d | 120 w  ±  7d |
| Written informed  consent | X |  |  |  |  |  |  |  |  |  |  |  |  |  |  |  |  |  |  |  |  |  |  |  |  |  |  |  |  |
| Collect medical  history | X |  |  |  |  |  |  |  |  |  |  |  |  |  |  |  |  |  |  |  |  |  |  |  |  |  |  |  |  |
| Physical  examination | X | X | X | X | X | X | X | X | X | X | X | X | X | X | X | X | X | X | X | X | X | X | X | X | X | X | X | X | X |
| Signs and symptoms | X | X | X | X | X | X | X | X | X | X | X | X | X | X | X | X | X | X | X | X | X | X | X | X | X | X | X | X | X |
| Blood  coagulation | X |  |  |  |  |  |  |  | X |  |  |  |  |  | X |  |  |  |  |  | X |  |  |  |  |  | X |  | X |
| HCG^1^ |  | X |  |  |  |  |  |  |  |  |  |  |  |  | X |  |  |  |  |  |  |  |  |  |  |  | X |  |  |
| Routine urine  test^2^ | X | X |  |  |  |  |  |  | X |  |  |  |  |  | X |  |  |  |  |  | X |  |  |  |  |  | X |  |  |
| Abdominal  ultrasound | X |  |  |  |  |  |  |  | X |  |  |  |  |  | X |  |  |  |  |  | X |  |  |  |  |  | X |  |  |
| ECG | X |  |  |  |  |  |  |  |  |  |  |  |  |  | X |  |  |  |  |  |  |  |  |  |  |  | X |  |  |
| Chest X ray | X |  |  |  |  |  |  |  |  |  |  |  |  |  | X |  |  |  |  |  |  |  |  |  |  |  | X |  |  |
| Ophthalmo-  logical  examination | X |  |  |  |  | X |  |  | X |  |  |  |  |  | X |  |  |  |  |  | X |  |  |  |  |  | X |  |  |
| AFP | X |  |  |  |  |  |  |  |  |  |  |  |  |  | X |  |  |  |  |  |  |  |  |  |  |  | X |  | X |
| Routine  Blood  test^3^ | X | X | X | X | X | X | X | X | X | X | X | X | X | X | X | X | X | X | X | X | X | X | X | X | X | X | X | X | X |
| biochemical indexes^4^ | X | X | X | X | X | X | X | X | X | X | X | X | X | X | X | X | X | X | X | X | X | X | X | X | X | X | X | X | X |
| HBeAg^5^ | X | X |  |  |  | X |  |  | X |  |  | X |  |  | X |  |  | X |  |  | X |  |  | X |  |  | X |  | X |
| HBsAg^5^ | X | X |  |  |  | X |  |  | X |  |  | X |  |  | X |  |  | X |  |  | X |  |  | X |  |  | X |  | X |
| HBsAb^5^ |  |  |  |  |  |  |  |  | X |  |  |  |  |  | X |  |  |  |  |  | X |  |  |  |  |  | X |  | X |
| HBV DNA^5^ | X | X |  |  |  | X |  |  | X |  |  | X |  |  | X |  |  | X |  |  | X |  |  | X |  |  | X | X |  |
| T3，T4，TSH | X |  |  |  |  | X |  |  | X |  |  | X |  |  | X |  |  | X |  |  | X |  |  | X |  |  | X |  | X |
| Fibroscan | X |  |  |  |  |  |  |  | X |  |  |  |  |  | X |  |  |  |  |  | X |  |  |  |  |  | X |  | X |
| ANA | X |  |  |  |  |  |  |  |  |  |  |  |  |  | X |  |  |  |  |  |  |  |  |  |  |  | X |  | X |
| HIV, HAV,  HCV, HDV,  HEV | X |  |  |  |  |  |  |  |  |  |  |  |  |  |  |  |  |  |  |  |  |  |  |  |  |  |  |  |  |
| Adverse events |  | X | X | X | X | X | X | X | X | X | X | X | X | X | X | X | X | X | X | X | X | X | X | X | X | X | X | X | X |
| Medication dispensed |  | X | X | X | X | X | X | X | X | X | X | X | X | X | X | X | X | X | X | X | X | X | X | X | X | X |  |  |  |
| Medication  returned |  |  | X | X | X | X | X | X | X | X | X | X | X | X | X | X | X | X | X | X | X | X | X | X | X | X | X |  |  |
| Serum sample | X | X |  | X |  | X |  |  | X |  |  | X |  |  | X |  |  | X |  |  | X |  |  | X |  |  | X | X | X |
| Whole  blood  sample | X | X |  | X |  | X |  |  | X |  |  | X |  |  | X |  |  | X |  |  | X |  |  | X |  |  | X | X | X |
| Immunologic parameters^6^ | X | X |  | X |  | X |  |  | X |  |  | X |  |  | X |  |  |  |  |  | X |  |  |  |  |  | X | X |  |
| PBMC^6^ | X | X |  | X |  | X |  |  | X |  |  | X |  |  | X |  |  | X |  |  | X |  |  | X |  |  | X | X | X |
| Liver biopsy |  | X |  |  |  |  |  |  |  |  |  |  |  |  | X |  |  |  |  |  |  |  |  |  |  |  |  |  | X |

^1^HCG testing: Only performed in female patients within 24 hours of the first dose of study drug;

1. Routine urine test includes pH value, red blood cell, white blood cell, urine sugar and proteinuria;
2. Routine blood test includeS hemoglobin, red blood cell counts, white blood cell counts, neutrophils, lymphocytes, monocytes, and platelet counts;
3. Blood biochemical indexes include ALT, AST, total bilirubin, total cholesterol, triglycerides, total protein, albumin, urea nitrogen, creatinine, serum potassium, calcium, phosphorus, blood glucose;
4. Quantitative HBsAg will be performed by central lab;
5. Immunologic parameters study and PBMC separation & freezing only performed in center of Tongji hospital.

## Screening examination and eligibility screening form

All legal guardians/subjects must sign and date the most current IRB/IEC-approved written informed consent/assent (where appropriate) before any study-specific assessments or procedures are performed.

The assessments in Table 2 must be obtained within a period of no more than 8 weeks before the subject is randomized, except as noted. Subjects must fulfill all the entry criteria for participation in the study (see Sections 6.2 to 6.3). An Eligibility Screening Form (ESF) documenting the investigator’s assessment of each screened subject with regard to the protocol’s inclusion and exclusion criteria is to be completed by the investigator. A screen failure log must be maintained by the investigator.

Table 2 Screening Assessments

| Medical history and physical examination | Includes family history of HBV, concomitant medication (including for CHB), and vital signs (blood pressure, heart rate, temperature). |
| --- | --- |
| Ophthalmological examination | By ophthalmologist and including fundoscopic examination, visual acuity assessment, visual field testing, and color visual testing. Any subject who develops ocular symptoms should receive a prompt eye examination by an ophthalmologist and additional examinations as necessary. |
| Ultrasound and liver elastography | To rule out hepatocellular carcinoma and liver cirhosis. Further liver imaging assessment may be performed if necessary. |
| Liver biopsy | Optional |
| HCG pregnancy test | For females of childbearing potential, a negative urine or serum HCG test needs to be documented within 24 hours prior to baseline. Note that the test may be performed at the baseline visit, provided that the result is available prior to randomization and commencement of treatment. |
| Hematology | Complete blood count (hemoglobin, hematocrit, total white blood cell [WBC] count, differential WBC count [neutrophils, lymphocytes, monocytes, eosinophils, basophils]), platelet count, international normalized ratio (INR) |
| Clinical chemistry | ALT, AST, GGT, total bilirubin, alkaline phosphatase, total |
|  | protein, albumin, blood urea nitrogen (BUN)/urea, creatininee, uric acid, total calcium, phosphorus, cholesterol, triglycerides, random glucose, sodium, chloride, potassium |
| Urinalysis | Dipstick with subsequent microscopic evaluation if positive for blood |
| Immunology and special chemistry | Alfa-fetoprotein, ANA, anti-HAV IgM, anti-HCV, anti-HIV, anti-  HDV, anti-HEV IgM |
| HBV serology and virology | HBeAg, anti-HBe, HBsAg, anti-HBs, quantitative HBsAg and  HBeAg, HBV-DNA |
| Thyroid function tests | FT3, FT4, TSH |

## Procedures for enrollment of eligible subjects

A subject will be enrolled in the study and assigned a screening number after the subject has given informed consent/assent. Once a subject has fulfilled the entry criteria they may be randomized or assigned, as applicable.

The subject randomization numbers will be generated by SAS PROC PLAN with a block size of 6 and were communicated to the sites in sealed envelopes prepared by the statistician based on the randomization sequence. The investigator or designee will use the CRF with the assigned subject number and enter the corresponding number for allocation to the study groups in the appropriate place on each subject’s CRF.

## Clinical assessments and procedures

A baseline visit will be performed, and for subjects in three groups all baseline assessments and procedures must occur prior to administration of the first dose of study drug. Subjects will subsequently be seen for evaluation at the following timepoints:

During treatment period: Day 1, Week 2, 4, 8, 12, 16, 20, 24, 28, 32, 36, 40, 44, 48, 52, 56, 60, 64, 68, 72, 76, 80, 84, 88, 92 and 96

During follow-up: Week 24 post-treatment

Any subject who discontinues study treatment should return to complete assessments as per the Week 96 visit.

### Efficacy

Efficacy (see Section 5.1 for primary and secondary endpoints) will be determined from assessment of loss of HBsAg, HBsAg positivity and seroconversion, HBeAg loss, HBV-DNA and ALT.

### Safety

Safety assessments will include symptom directed physical examination, vital signs, review of concomitant medications, reported AEs and specified safety laboratory assessments of hematology, clinical chemistry, thyroid function, and urinalysis.

Additional safety assessments will be determined from premature withdrawals from treatment for safety or tolerability reasons.

All subjects need to have ophthalmological examination (including fundoscopic examination, visual acuity assessment, visual field testing, and color visual testing) by an ophthalmologist. Thereafter, subjects treated with IFN will receive ophthalmological examination as per the schedules in Table 1. Any subject who develops ocular symptoms should receive a prompt eye examination by an ophthalmologist and additional examinations as necessary.

For females of childbearing potential, a pregnancy test will be performed within 24 hours prior to baseline. This could be performed at the baseline visit, provided that the result is available prior to randomization and commencement of treatment. In addition, subjects will have pregnancy tests performed as per the schedules in Table 1.

## Laboratory assessments

Laboratory assessments post-screening are noted in Table 3 and will be carried out as per the schedules in Table 1.

Table 3 Laboratory Assessments

| Hematology | Complete blood count (hemoglobin, hematocrit, total white blood cell [WBC] count, differential WBC count [neutrophils, lymphocytes, monocytes, eosinophils, basophils]), platelet count, international normalized ratio (INR) |
| --- | --- |
| Clinical chemistry | ALT, AST, GGT, total bilirubin, alkaline phosphatase, total protein, albumin, blood urea nitrogen (BUN)/urea, creatininee, uric acid, total calcium, phosphorus, cholesterol, triglycerides, random glucose, sodium, chloride, potassium |
| Urinalysis | Dipstick with subsequent microscopic evaluation if positive for blood |
| Immunology and special chemistry | Alfa-fetoprotein, ANA |
| HBV serology and virology | HBeAg, anti-HBe, HBsAg, anti-HBs, quantitative HBsAg and  HBeAg, HBV-DNA |
| Thyroid function tests | FT3, FT4, TSH |
| HCG pregnancy test | For females of childbearing potential, subjects will have pregnancy tests performed as per the schedules in Table 1. |

HBsAg will be assessed by the central laboratory. Other parameters will be assessed by a local laboratory, normal ranges for the local laboratory parameters must be supplied to designee before the study starts and at any point that the normal ranges are changed.

## Subject medication diary review

Subjects will complete a Subject Medication Diary (SMD), which the patients will bring to each study visit. The information will be reviewed by the Clinic Coordinator at each study visit. It is mandatory that each drug dose be recorded in the SMD. The patient/guardian will be instructed to record the date and dose of study drug. The person giving the medication must initial the SMD.

During the diary review, the reviewer will correct unreadable information and ask the subject to supply missing information. Reviewers will report missing doses, dose adjustments, and treatments stops or restarts on the CRF. Subject diaries will be maintained with the source documents at each site.

## Concomitant medication and treatment

Use of any investigational drugs as a result of participation in another clinical study is prohibited during the study including the extended long-term follow-up period.

Concomitant use of any other investigational drugs (e.g., compassionate use), immunomodulatory treatments (e.g., systemic corticosteroids), growth factors (e.g., erythropoietin) or antiviral treatments with anti-HBV activity (e.g., lamivudine, tenofovir, emtricitabine, adefovir, telbivudine, systemic acyclovir, systemic famciclovir) other than those specifically allowed by this protocol are prohibited during either the treatment period or follow-up, in order to minimize the confounding effects of such treatments in determining treatment responses.

Exceptions to this are as follows:

Subjects with significant deterioration in hepatic function who permanently discontinue study drug and need to consider NA therapies in group II and group III, treatments with anti-HBV activity will not be prohibited during follow-up period.

Herbal, botanical, and other agents that are traditionally used for chronic HBV disease are discouraged.

All concomitant medications should be reported to the investigator and recorded on the appropriate electronic Case Report Form (CRF), including details of any previous anti-HBV treatment, as well as any other previous concomitant medications taken within 6 months

prior to baseline.

## Criteria for premature withdrawal

Subjects have the right to withdraw from study treatment at any time for any reason.

In the case that the subject decides to prematurely discontinue study treatment (“refuses treatment”), all efforts will be made to complete and report the observations prior to withdrawal as thoroughly as possible preferably by returning to site to complete assessments as per the Week 96 visit and the Treatment Completion CRF.

If possible, a complete final evaluation at the time of the subject’s withdrawal from the study should be made with an explanation of why the subject is withdrawing from the study. When applicable, subjects should be informed of circumstances under which their participation may be terminated by the investigator without the subject’s consent/assent. The investigator may withdraw subjects from the study in the event of intercurrent illness, AEs, pregnancy, lack of compliance with the study and/or study procedures (e.g., dosing instructions, study visits) or any reason where it is felt by the investigator that further follow-up of the subject is impossible or it is in the best interest of the subject to be terminated from the study. Any administrative or other reasons for withdrawal must be documented and explained to the subject.

If the reason for removal of a subject from the study is an AE, the principal specific event will be recorded on the CRF. The subject should be followed until the AE has resolved, if possible.

An excessive rate of withdrawals can render the study non-interpretable; therefore, unnecessary withdrawal of subjects should be avoided.

# Schedule of investigation medical product

## Stopping rules

Individual subject will be stopped in the event of any of the following:

- Severe hypersensitivity reactions (e.g., anaphylaxis, angioedema, bronchoconstriction) • Evidence of hepatic decompensation, e.g., significant deterioration in hepatic function including those requiring alternative therapies
- Severe depression
- Convulsions
- Thyroid abnormalities that cannot be adequately treated
- Hypoglycemia, hyperglycemia, or diabetes mellitus that cannot be effectively controlled by medication
- New or worsening visual disorders such as field deficits, decreased or loss of vision
- Persistent or unexplained pulmonary infiltrates or pulmonary function impairment
- Worsening of psoriatic lesion
- Development of autoimmunity, including autoimmune hepatitis
- Renal failure, including creatinine >100 μmol/L
- Severe symptoms e.g., rigors
- Absolute neutrophil count < 0.25 × 10 ^9^cells/L or febrile neutropenia
- Platelets < 25 × 10 ^9^ cells/L
- Pregnancy

## Missing Consecutive Doses

If four or more consecutive doses of study drugs are held or otherwise not administered (i.e., the subject has not received study drug for more than 28 days), the subject will be considered intolerant of the study drug or noncompliant, whichever is more appropriate to the clinical situation. No additional study drug may be administered to such subjects without explicit permission from the Sponsor.

## Accountability of study medicinal product

The investigator is responsible for the control of drugs under investigation. Adequate records for the receipt (e.g., Drug Receipt Record) and disposition (e.g., Drug Dispensing Log) of the study drug must be maintained. Accountability will be assessed by maintaining adequate drug dispensing and return records.

Accurate records must be kept for each study drug. These records must contain the

following:

- Documentation of drug shipments (date received and quantity).
- Disposition of unused study drug not dispensed to subject.

Study drug will be dispensed according to the schedules in Table 1. A Drug Dispensing

Log must be kept current and should contain the following information:

- The date(s) and quantity of the study drug dispensed to the subject
- The date(s) and quantity of the study drug returned by the subject

All records and drug supplies must be available for inspection by the Monitor at every

monitoring visit.

Subjects will be asked to return all used drug supply containers at each visit and all unused drug supply containers at the end of treatment as a measure of compliance.

## Assessment of Compliance

At each visit, compliance with the study requirements will be reinforced.

Subject compliance with taking study drug as required will be assessed by maintaining adequate study drug dispensing records and review of the SMD. The investigator is responsible for ensuring that dosing is administered in compliance with the protocol.

Delegation of this task must be clearly documented and approved by the investigator.

It is mandatory that each administration (date and dose) be recorded in the drug diary. Subjects will be asked to return used drug containers at each visit, along with the drug diary. Returned drug containers will be counted and justified against the diary entries by study site personnel. Each verified administration will be recorded on the CRF at each visit. Unused study drug should be returned at the end of the treatment period. Under no circumstances should the treatment period for any subject extend beyond the allocated duration.

## Destruction of the study medicine

Local or institutional regulations may require immediate destruction of used study medication for safety reasons. In these cases, it may be acceptable for investigational site staff to destroy dispensed drugs before a monitoring inspection provided that source document verification is performed on the remaining inventory and reconciled against the documentation of quantity shipped, dispensed, returned, and destroyed. Written authorization must be obtained at study start up before destruction.

# Safety instruction and guidance

## Insurance and indemnity

All subjects participating in this trial will be covered for any trial medicine-related adverse effects through insurance cover to be taken out by the Sponsor.

## Adverse events and laboratory abnormalities

### Clinical adverse events

According to the ICH, an AE is any untoward medical occurrence in a subject or clinical investigation subject administered a pharmaceutical product and which does not necessarily have a causal relationship with this treatment. An AE can therefore be any unfavorable and unintended sign (including an abnormal laboratory finding), symptom, or disease temporally associated with the use of a medicinal (investigational) product, whether or not considered related to the medicinal (investigational) product. Preexisting conditions that worsen during a study are to be reported as AEs.

### Intensity

All clinical AEs encountered during the clinical study will be reported on the AE CRF.

Intensity of AEs will be graded (mild, moderate, severe) and reported in detail on the CRF.

| Mild | discomfort noticed but no disruption of normal daily activity |
| --- | --- |
| Moderate | discomfort sufficient to reduce or affect daily activity |
| Severe | inability to work or perform normal daily activity |

Note that events that are considered as life threatening should be reported as an SAE and the intensity would be reported as “severe” on the CRF.

### Drug-Adverse Event Relationship

Investigators should use their knowledge of the subject, the circumstances surrounding the event, and an evaluation of any potential alternative causes to determine whether or not an adverse event is considered to be related to the study drug, indicating "yes" or "no" accordingly. The following guidance should be taken into consideration:

- Temporal relationship of event onset to the initiation of study drug
- Course of the event, considering especially the effects of dose reduction, discontinuation of study drug, or reintroduction of study drug (where applicable)
- Known association of the event with the study drug or with similar treatments
- Known association of the event with the disease under study
- Presence of risk factors in the subject or use of concomitant medications known to increase the occurrence of the event
- Presence of non-treatment-related factors that are known to be associated with the occurrence of the event

### Serious Adverse Events (Immediately Reportable to Principal Investigator)

An SAE is any experience that suggests a significant hazard, contraindication, side effect, or precaution. It is any AE that at any dose fulfils at least one of the following criteria:

- Is fatal (results in death**; note: death is an outcome, not an event)
- Is life-threatening (Note: the term “life-threatening” refers to an event in which the subject was at immediate risk of death at the time of the event; it does not refer to an event which could hypothetically have caused a death had it been more severe)
- Requires in-patient hospitalization or prolongation of existing hospitalization
- Results in persistent or significant disability/incapacity
- Is a congenital anomaly/birth defect
- Is medically significant or requires intervention to prevent one or other of the outcomes listed above.

**The term sudden death should be used only when the cause is of a cardiac origin as per standard definition. The terms death and sudden death are clearly distinct and must not be used interchangeably.

The study will comply with all local regulatory requirements and adhere to the full requirements of the ICH Guideline for Clinical Safety Data Management, Definitions and

Standards for Expedited Reporting, Topic E2.

### Treatment and Follow-up of Adverse Events

Subjects experiencing AEs should be treated by accepted clinical procedures. If the use of medications excluded by protocol is deemed necessary, the subject may need to be discontinued from the test regimen after consultation with principal investigator. All AEs should be followed until resolved or stabilized.

### Laboratory Test Abnormalities

Laboratory test results will be recorded on the laboratory results form page of the CRF. Any laboratory result abnormality fulfilling the criteria for an SAE should be reported as such on the AE Form.

Any treatment-emergent abnormal laboratory result which is clinically significant, i.e., meeting one or more of the following conditions, should be recorded as a single diagnosis on the AE Form in the CRF:

- Is considered an SAE
- Results in discontinuation from study treatment
- Results in a requirement for a change in concomitant therapy (e.g., addition of, interruption of, discontinuation of, or any other change in a concomitant medication, therapy or treatment)

This applies to any protocol and non-protocol-specified safety and efficacy laboratory result from tests performed after the first dose of study drug, which falls outside the laboratory reference range and meets the clinical significance criteria.

The finding of an elevated ALT or AST (>3 × baseline value) in combination with either an elevated total bilirubin (>2 × ULN) or clinical jaundice, in the absence of cholestasis or other causes of hyperbilirubinemia, is considered to be an indicator of severe liver injury. Therefore, investigators must report as an AE the occurrence of either of the following:

- Treatment-emergent ALT or AST>3 × baseline value in combination with total bilirubin >

2 × ULN (of which 35% is direct bilirubin) or

• Treatment-emergent ALT or AST >3 × baseline value in combination with clinical jaundice The most appropriate diagnosis (or if a diagnosis cannot be established, the abnormal laboratory values) should be recorded on the AE CRF and reported to the principal investigator immediately (i.e., no more than 24 hours after learning of the event), either as an SAE or a non-serious AE of special interest.

### Follow-up of Abnormal Laboratory Test Values

Clinically significant laboratory abnormalities should prompt a repeat measure no less frequently than every 2 weeks or as clinically indicated, with appropriate clinical management, until they return to normal or baseline levels, and/or an adequate explanation of the abnormality is found. If a clear explanation is established it should be recorded on the CRF.

## Handling of safety parameters

### Reporting of Adverse Events

All AEs (related and unrelated) occurring during the treatment and follow-up must be reported.

### Reporting of Serious Adverse Events (immediately reportable)

Any clinical AE or abnormal laboratory test value that is serious and that occurs during the study, regardless of the treatment arm, occurring from the enrollment visit (start of study screening procedures), including follow-up must be reported to principal investigator within 1 working day of the investigator becoming aware of the event.

For initial reports of serious adverse events, investigators should record all case details that can be gathered within 1 working day on the Adverse Event CRF. A report will be generated and sent to principal investigator. A paper Serious Adverse Event CRF and Fax Cover Page should be completed and faxed immediately to principal investigator.

After informed consent, but prior to initiation of study medications, only SAEs caused by a protocol-mandated intervention will be collected (e.g., SAEs related to invasive procedures such as biopsies, medication washout, or no treatment run-in). After first study drug, all SAEs must be reported.

Unrelated SAEs must be collected and reported during the study treatment and follow-up. Related SAEs must be collected and reported regardless of the time elapsed from the last study drug administration, even if the study has been closed. Suspected Unexpected Serious Adverse Reactions (SUSARs) are reported to investigators at each site and associated IRB/IEC when the following conditions occur:

- The event must be an SAE.
- There must be a certain degree of probability that the event is an adverse reaction from the administered drug.
- The adverse reaction must be unexpected.

This study adheres to the definition and reporting requirements of ICH Guideline for Clinical

Safety Data Management, Definitions and Standards for Expedited Reporting, Topic E2.

### Pregnancy

Subjects must utiliz reliable contraception during the treatment and during follow-up period. A female subject must be instructed to stop taking the study medication and immediately inform the investigator if she becomes pregnant during the study. The investigator should report all pregnancies within 24 hours to the principal investigator/designee, using the Pregnancy Report CRF. The investigator should counsel the subject, and discuss the risks of continuing with the pregnancy and the possible effects on the fetus. Monitoring of the subject should continue until conclusion of the pregnancy. Pregnancies occurring up to 90 days after the completion of the study medication must also be reported to the investigator. Subjects that become pregnant during the treatment period will be withdrawn from the treatment period.

Pregnancy occurring in the partner of a male subject participating in the study should be reported to the investigator and principal investigator/designee. The partner should be counseled, the risks of continuing the pregnancy discussed, as well as the possible effects on the fetus. Monitoring of the subject should continue until conclusion of the pregnancy. A Pregnancy Report worksheet and Pregnancy Fax Coversheet should be completed and faxed to principal investigator/designee within 1 working day after learning of the pregnancy.

### Warnings and precautions

IFN may cause or aggravate fatal or life-threatening neuropsychiatric, autoimmune, ischemic, and infectious disorders. Subjects should be monitored closely with periodic clinical and laboratory evaluations. Therapy should be withdrawn from subjects with persistently severe or worsening signs or symptoms of these conditions. In many, but not all cases, these disorders resolve after stopping IFN therapy.

There are no adequate, controlled studies of any IFN in pregnant females. Therefore, extreme care must be taken to avoid pregnancy during the study in female subjects, and female partners of male subjects taking IFN. Females who are pregnant or breast-feeding will be excluded. A pregnancy test will be performed for each female of childbearing potential prior to baseline and be performed according to the schedules in Table 1.

# The complete statistical analysis plan

## Primary and secondary study endpoints

### Primary endpoints

The primary efficacy parameter is HBsAg loss as measured by the Roche Elecsys HBsAg II Quant assay (Roche Diagnostics, Penzberg, Germany; dynamic range 0.05-52,000 IU/ml), with both taken at end of treatment and 24 weeks after the end of treatment.

### Secondary endpoints

The secondary efficacy parameters include (at end of treatment and 24 weeks after the end of treatment)

1. HBsAg seroconversion
2. Proportion of patients who achieve HBV DNA < 1000 copies/ml
3. Quantitative HBsAg measurement
4. HBeAg seroconversion
5. ALT normalization

Histology improvement (option)

### Safety

Safety of the treatment will be evaluated by AEs (including SAEs), laboratory tests, vital signs (blood pressure, heart rate, temperature).

## Statistical methods

### Primary Variables

The primary efficacy parameter is HBsAg loss at end of treatment and 24 weeks after the end of treatment. Subjects with missing values for this parameter will be considered non-responders.

### Secondary Variables

All secondary variables will be determined at end of treatment and 24 weeks after the end of treatment.

1. HBsAg positivity (defined as presence of anti-HBs) and seroconversion (defined as loss of HBsAg and presence of anti-HBs)
2. Proportion of patients who achieve undetectable HBV DNA
3. Quantitative HBsAg measurement
4. HBeAg seroconversion (defined as absence of HBeAg and presence of HBeAb in

HBeAg negative patients)

1. ALT normalization (defined as ALT measure ≤ ULN)
2. Sustained virologic response at the end of follow-up

Histology improvement (option)

### Sample Size

It was assumed that the HBsAg loss rate would be 3-10% in the IFN arm based on OSST study in NA-suppressed patients, and that HBsAg loss rate in ETV arm would be 0-3%.

But there is a lack of data regarding HBsAg loss rate by the novel treatment strategy using IFN in combination with GM-CSF. As a pilot and a proof of concept study, the sample size was set at approximate 80-100 per group to investigate whether this combination therapy could induce higher HBsAg loss.

### Analysis populations: ITT and mITT population

The ITT population shall include all subjects who receive at least one dose of study medication. For the ITT population, subjects will be analyzed according to the groups to which they were randomized.

A modified ITT (mITT) analysis was conducted, excluding patients who failed to meet inclusion criteria at baseline prior to randomization. Any patients including in mITT analysis who withdrew from the study, for whatever reason, was classified as a non-responder.

### Efficacy Analysis

The primary and secondary efficacy analysis will compare efficacy among three groups.

The descriptive statistics depending on the type of variables will be as follows:

Quantitative variable: size, mean, standard deviation, minimum and maximum.

Qualitative, ordinal variables: frequency and percentage per class.

Response rates for primary and secondary endpoints were assessed by calculating percentages and 95% confidence intervals (CIs) for the ITT and mITT populations. Categorical variables were analyzed by the v2 test or Fisher’s exact test, and continuous variables were analyzed by t test or Wilcoxon test as appropriate. Logarithmic transformation was performed in the case of skewed data. Statistical analyses were conducted by SAS version 9.2.

### Exclusion of Data from Analysis

#### Intent-to-Treat Population and modified ITT Population

All subjects randomized will be included in the ITT population.

A modified ITT (mITT) analysis was conducted, excluding patients who failed to meet

inclusion criteria at baseline prior to randomization.

Subjects will be assigned to groups as randomized for analysis purposes.

Subjects will be excluded if they do not meet mITT criteria.

#### Safety Data Analysis

The primary safety analysis will compare safety among three groups.

AEs: AEs will be assigned preferred terms and categorized into body systems according to the Medical Dictionary for Regulatory Activities (MedDRA) classification of the World Health Organization (WHO) terminology. Descriptive statistics will be used to summarize safety parameters by group.

Laboratory Safety Data: The laboratory data (including thyroid function) will be analyzed.

# Data collection, management, and assurance

Data for this study will be recorded in CRF. In no case is the CRF to be considered as source data for this trial except where stated in this protocol. Sites will receive training and a manual for appropriate CRF completion. Accurate and reliable data collection will be assured by verification and cross-check of the CRFs against the investigator’s records by the study monitor, and the maintenance of a drug dispensing log by the investigator.

Throughout the study the Study Management Team (SMT) will review data.
